# Modifications in the piperazine ring of nucleozin affect anti-influenza activity

**DOI:** 10.1371/journal.pone.0277073

**Published:** 2023-02-10

**Authors:** Erick Correa-Padilla, Alejandro Hernández-Cano, Gabriel Cuevas, Yunuen Acevedo-Betancur, Fernando Esquivel-Guadarrama, Karina Martinez-Mayorga

**Affiliations:** 1 Institute of Chemistry, National Autonomous University of Mexico, Mexico City, Mexico; 2 Zaragoza School of Higher Education, National Autonomous University of Mexico, Mexico City, Mexico; 3 School of Science, National Autonomous University of Mexico, Mexico City, Mexico; 4 School of Medicine, Autonomous University of the State of Morelos, Cuernavaca, Morelos, México; 5 Institute of Chemistry, Campus Merida, National Autonomous University of Mexico, Merida-Tetiz Highway, Yucatán, México; 6 Institute for Applied Mathematics and Systems, Merida Research Unit, National Autonomous University of Mexico, Sierra Papacal Merida, Yucatan, Mexico; Government College University Faisalabad, PAKISTAN

## Abstract

The infection caused by the influenza virus is a latent tret. The limited access to vaccines and approved drugs highlights the need for additional antiviral agents. Nucleozin and its analogs have gain attention for their promising anti-influenza activity. To contribute to the advancement of the discovery and design of nucleozin analogs, we analyzed piperazine-modified nucleozin analogs to increase conformational freedom. Also, we describe a new synthetic strategy to obtain nucleozin and its analogues, three molecules were synthesized and two of them were biologically evaluated *in vitro*. Although the analogues were less active than nucleozin, the loss of activity highlights the need for the piperazine ring to maintain the activity of nucleozin analogs. Interestingly, this result agrees with the prediction of anti-influenza activity made with a QSAR model presented in this work. The proposed model and the synthetic route will be useful for the further development of nucleozin analogs with antiviral activity.

## Introduction

Influenza is a highly contagious viral infection caused by a single-stranded negative-sense RNA virus of the *Orthomyxoviridae* family. This infection affects the respiratory system of the host, affecting the nasal pharyngeal mucosa, bronchi, and pulmonary alveoli. The symptoms of influenza are similar to the common cold; however, influenza can be deadly, especially in vulnerable groups. The mutation rate of the influenza virus; the high frequency of genetic rearrangement; and the antigenic changes in viral glycoproteins challenge the control of infections, causing even zoonotic interactions, such as avian influenza (H7 and H9) and swine influenza (Cal/09), with high potential for a pandemic threat. Pharmacological therapy is available. Rimantadine is used to treat or prevent the infection of seasonal influenza (Influenza B), and oseltamivir, peramivir, zanamivir are preferred for influenza (influenza A) that has reached epidemic and pandemic levels [1]. Furthermore, emergent strains are potentially life-threatening illnesses. Therefore, fostering the discovery and development of new therapeutic antiviral agents is paramount. Efforts in that direction led to the discovery of nucleozin (nlz), a potent inhibitor of influenza A virus infections in *in vitro* and *in vivo* assays [2,3]. Nlz, a piperazine amide, and the corresponding analogs induce the aggregation of nucleoprotein (NP), a protein that plays an essential role on the virus replication cycle. Three-dimensional structures, obtained by X-ray crystallography, of nlz and the analog named Gerritz 3 bound to NP, have been reported in the literature, **PDB ID: 3RO5** [4] and **PDB ID: 5B7B** [5], respectively. In the case of Gerritz 3, the three-dimensional structure shows a dimeric complex formed by two NP monomers bridged symmetrically by two molecules of compound Gerritz 3 [2(Gerritz 3):2NP]. In turn, the complex with nlz is belonging formed by six NP monomers and two nlz molecules (2nlz:6NP). These complexes precipitate in the nucleus of the host cell. They do not migrate to the cytoplasm, where it is necessary to form ribonucleoprotein (RNP) and the subsequent assembly of viral structures.

Influenza inhibitors targeting viral RNP constituent proteins have been recently reviewed [6,7]. Among the most promising targets is described NP, being **nlz** the representative inhibitor of this system. Recent strategies involve computational methodologies, such as virtual screening, similarity searches, and pharmacophore modeling.

Notably, **nlz** is an influenza inhibitor (IC_50_ = 0.06 μM) [2], more potent than oseltamivir (IC_50_ = 1–10 μM), an approved drug for the treatment of influenza infections [8]. In addition, the median toxic concentration (TC_50_) of **nlz** and analogs is greater than 250 μM. Thus, **nlz** and analogs are good drug candidates with a wide therapeutic window. To contribute to this area, here we report a novel synthesis of three **nlz** analogs, and the *in vitro* evaluation of two of those molecules. In addition, considering these molecules and **nlz** analogs reported in the literature, we present the development of predictive models of activity.

## Materials and methods

### QSAR models

**Fig 1:**
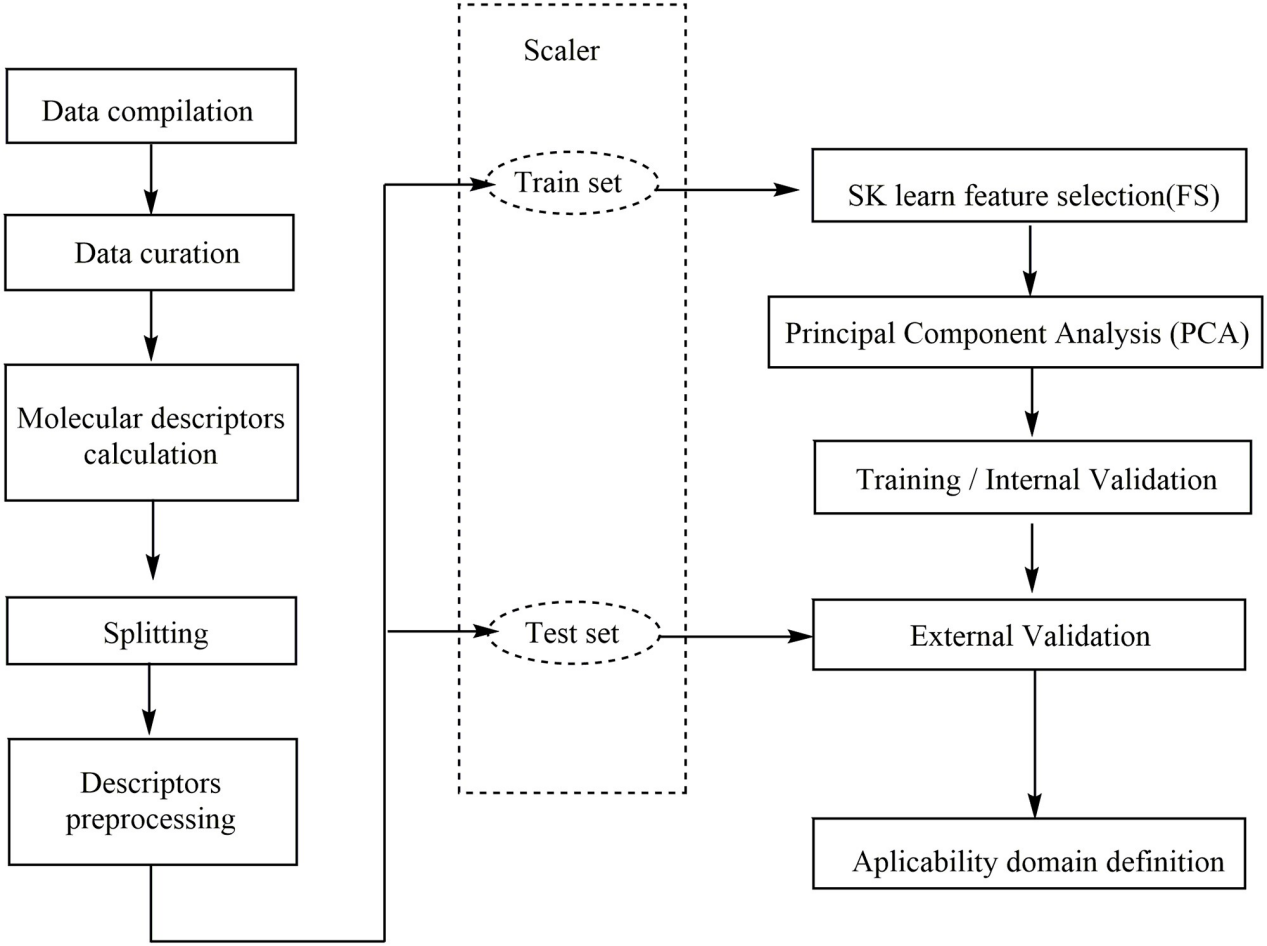
Workflow of the QSAR model.

The workflow to develop predictive models of **nlz** analogs with antiviral activity is shown in Fig 1 and consists of the following steps. Associated script can be found in the Supporting Information.

### Data compilation

The structures of **nlz** analogs were collected from the literature using the keyword “*nucleozin”* in SciFinder. The noncurated database consisted of 105 molecules; after the removal of datapoints without activity values, a final set contained 74 molecules. The biological activity reported for all these molecules was evaluated in the Plaque Reduction Assay (PRA) with the A/H1N1/WSN/33 strain [2,4,9]. The antiviral IC_50_ values were transformed to molar units and then to -log(IC_50_); this data is presented in supporting information in S1 Table in S1 File. ChemBioDraw Ultra 13.0 [10] was used to build the structures. Protonation states were assigned at pH = 7.0, and the structures were energy minimized with FFMM94x forcefield in MOE 2022.10 [11,12].

#### Molecular descriptors

4185 2D and 3D molecular descriptors were calculated with the software DRAGON (version 7) [13]. Eight additional descriptors (SLogP, Log S, lip_acc, lip_don, TPSA, Weight, b_rotN, b_rotR) were calculated with MOE 2019.10. and analyzed to assess ADME properties.

#### Feature selection and model training

The dataset (.csv file format) containing the molecular descriptors and activity values was imported into a notebook in Google Colaboratory (Colab) [14,15]. The modules NumPy [16], Matplotlib [17], Pandas [18,19] and Sci-kit Learn [20] were used for data handling, analysis, visualization, and for the generation of the supervised machine learning regression models. First, the data was split into training and test sets using a stratified split. The training and test sets contain 55 and 19 molecules, respectively. The descriptors with variance equal to cero, were removed with the algorithm VarianceThreshold, then, the descriptors were scaled with the STDScaler. Then, 70 descriptors relevant to the activity prediction were filtered using the Feature Selection (FS) algorithm from scikit-learn, using the meta transformer SelectFromModel, and the RandomForest Regressor as a base estimator. The feature selection step was next, using Principal Component Analysis (PCA) with 16 Principal Components and SVR (Support Vector Regressor) as the algorithm of regression for the training.

#### Model validation

Goodness-of-fit was measured with the coefficient of determination r^2^. Cross-Validation leave-many-out with 10 folds (CV-LMO, folds = 10), q^2^_CV-LMNO_, and Y-scrambling were performed to evaluate the robustness of the model. Lastly, the external validation, q^2^_ext_, allowed us to assess the predictive power.

### Synthesis of nucleozin and analogs

**Fig 2:**
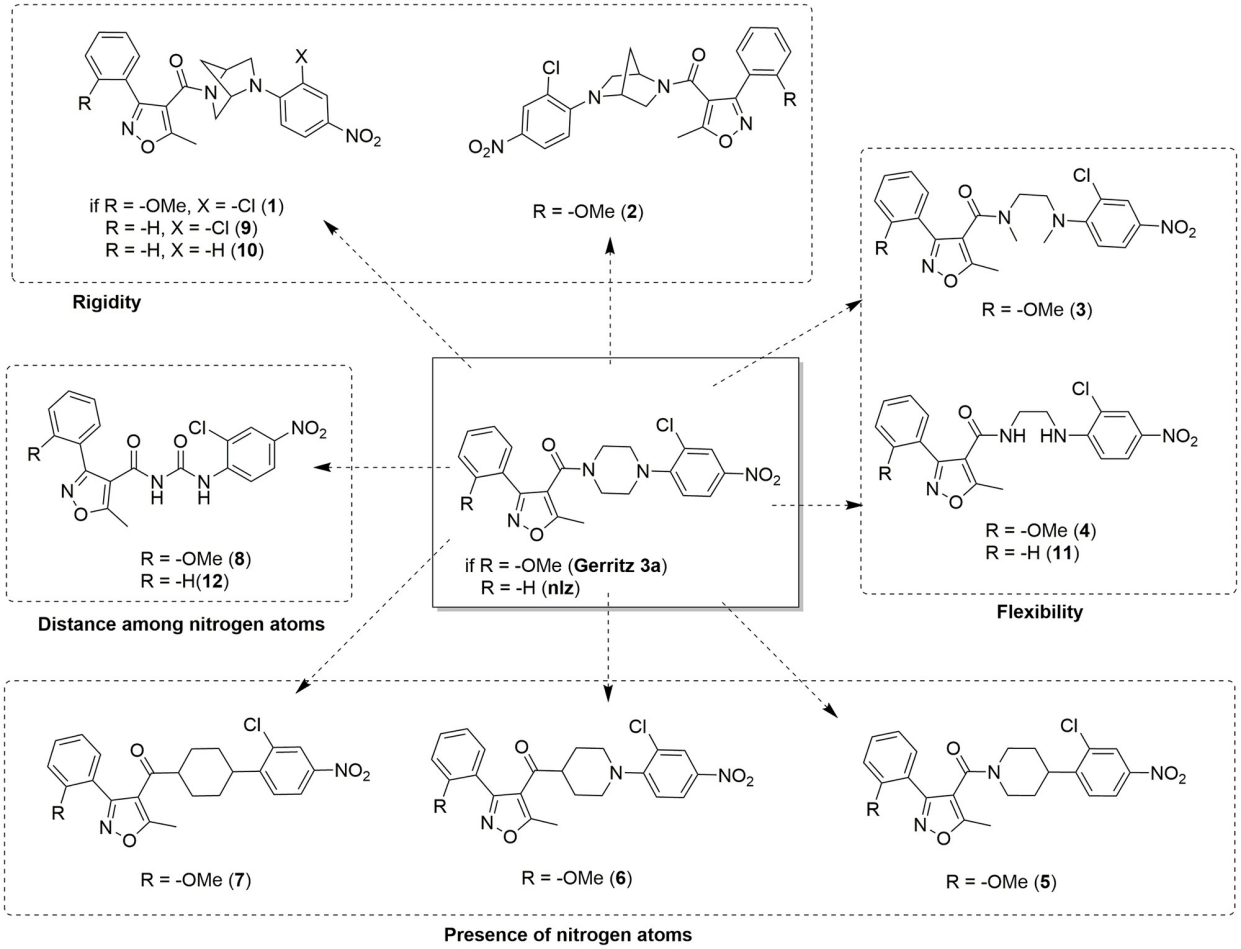
Analogs proposed in this work to study the influence of the piperazine moiety over the antiviral activity within the nucleozin derivatives.

**Fig 3:**
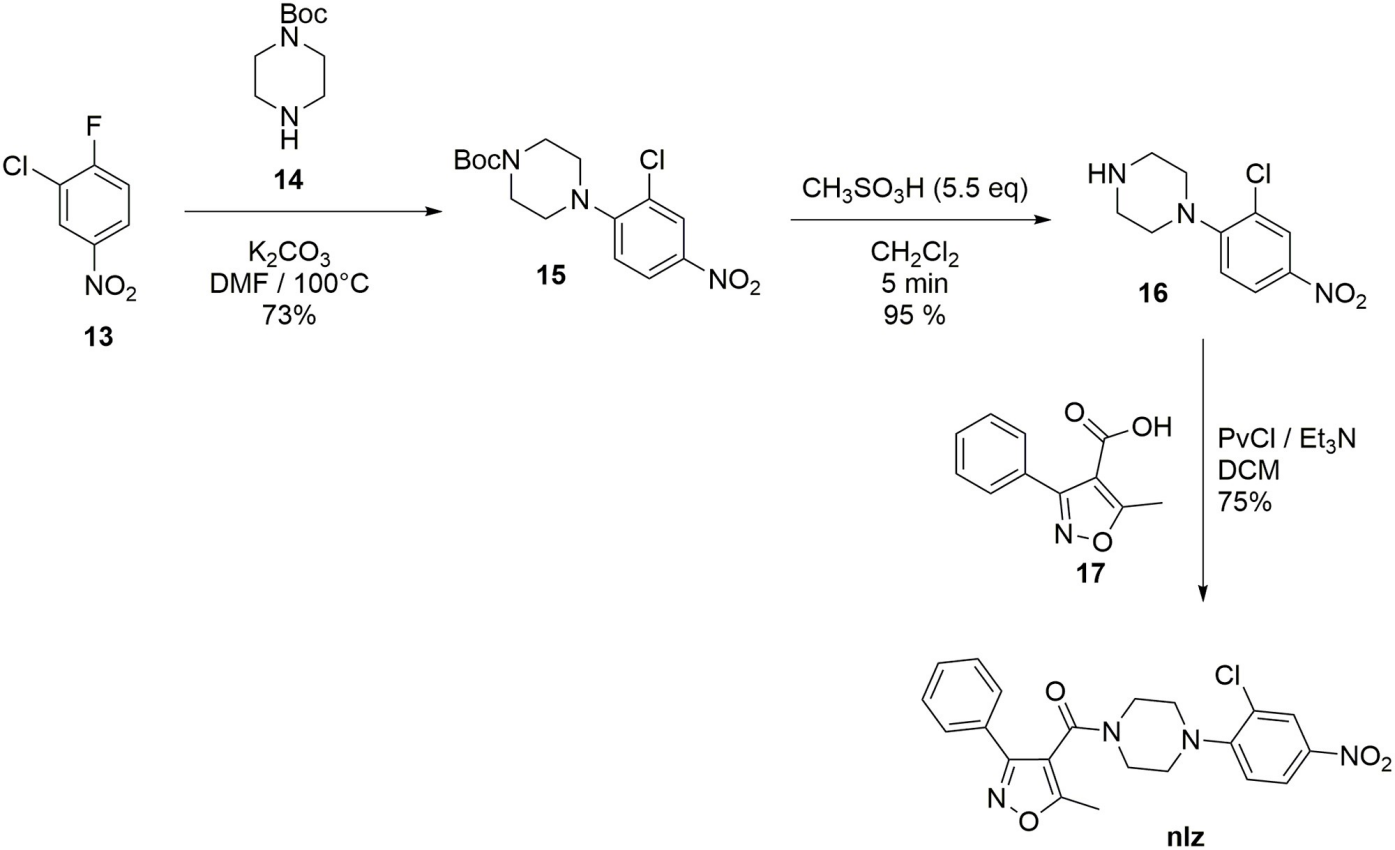
Synthesis of nucleozin.

**Fig 4:**
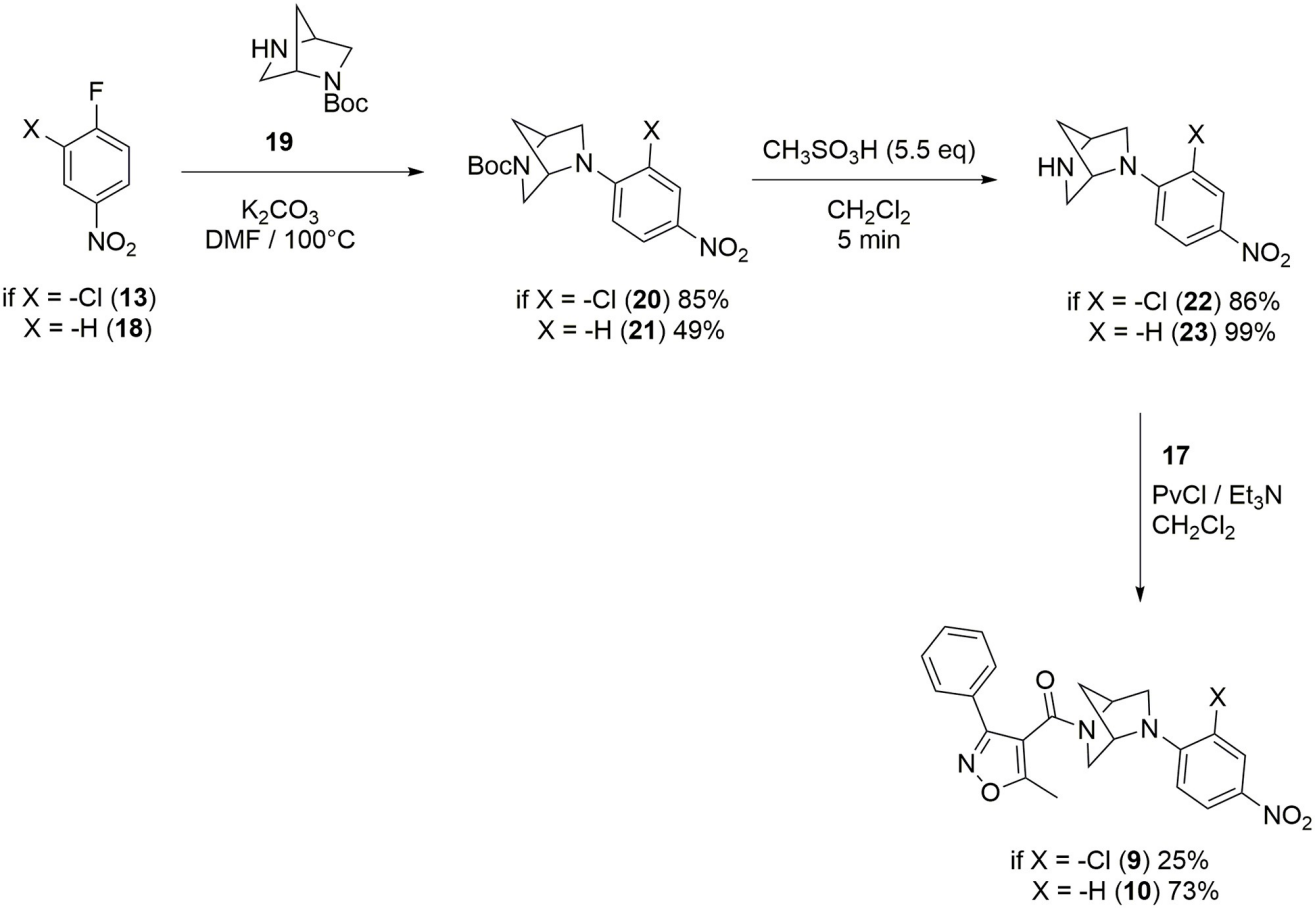
Synthesis of compounds 9 and 10.

**Fig 5:**
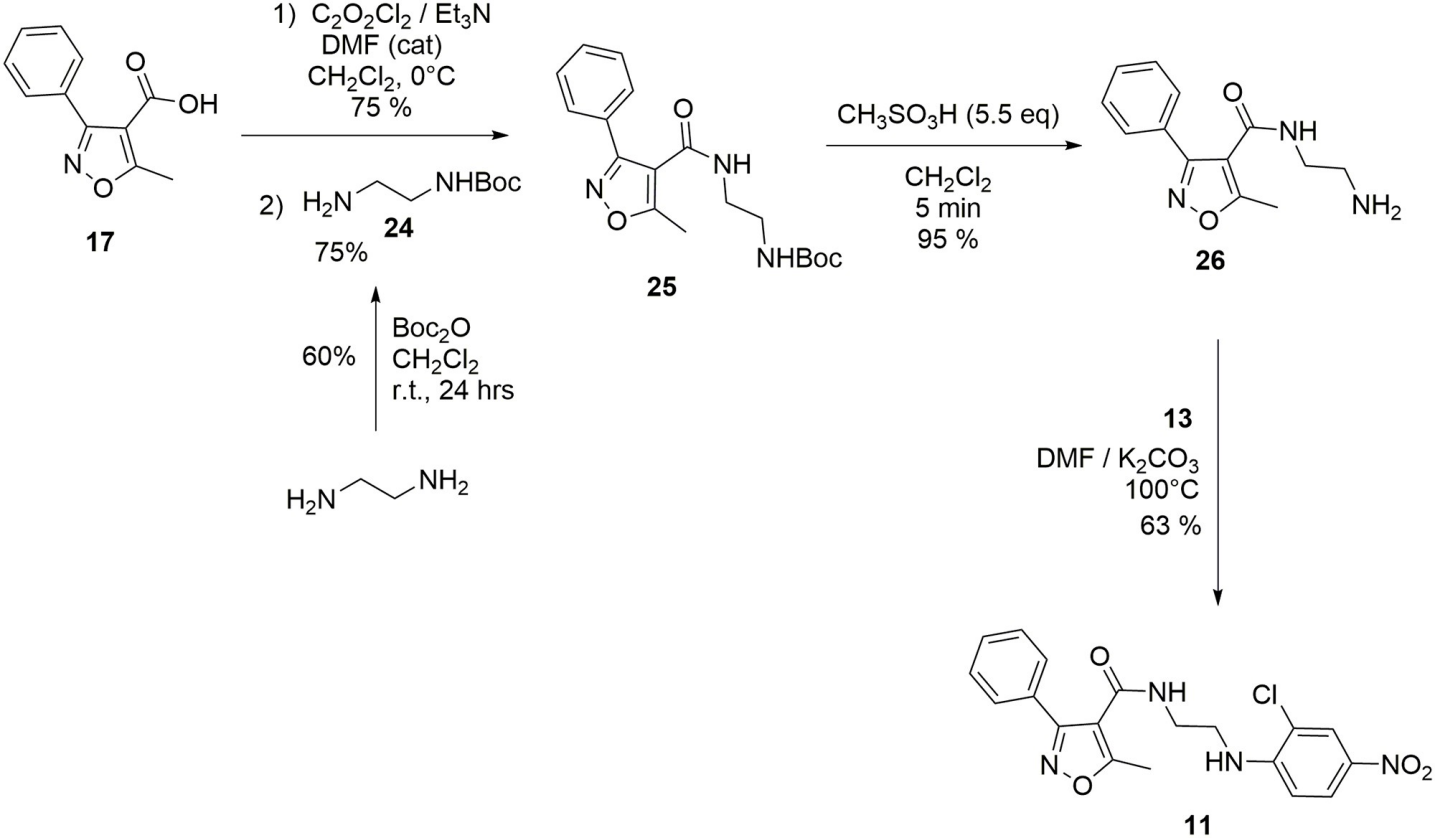
Synthesis of compound 11.

The chemical structures and numbering of the **nlz** analogs studied in this work are shown in Figs 2–5 and described in the Results section and the details of the synthesis can be found in the Supporting information section.

### Biological evaluation

A modified antigen-reduction assay was used to determine the activity nlz and their analogues [21]. Madin-Darby canine kidney (MDCK) cells were seed at 104 cells per well, in 96-well plates, in Dulbecco’s Modified Eagle’s Medium (DMEM) supplemented with 4 mM -L-glutamine, 1 mM sodium pyruvate, 50 U/mL penicillin, 50 μg/mL Streptomycin and 10% fetal calf serum (FCS), and incubated for 24 hr at 37 °C. The medium was retired, and cells infected for 1 hr a 37 °C with the influenza A virus strain A/Caledonia H1N1, at a concentration of 0.001 plaque forming units (PFU) per cell. The inoculum was retired, and different concentrations of **nlz** or their analogues (from 1 μM to 10 μM), diluted in DMEM without serum, added to the cells, and incubated. After 48 hrs, the supernatants were removed, frozen at -20 °C and then titrated in a new 96-well plate with MDCK and incubated for 1 hr at 37 °C. The inoculum was retired, DMEM without serum added to the cells and incubated for 72 hrs at 37 °C. The medium was retired, the cells fixed with 4% paraformaldehyde and permeabilized with methanol-acetone 1:1. Then, an ELISA on the fixed-permeabilized cells was performed, using a mouse monoclonal antibody specific for the internal viral protein M1 (ATCC; HB-64), followed by a secondary polyclonal antibody goat anti-mouse Ig’s conjugated with horseradish peroxidase (HRP) (Jackson). The reaction was developed with the substrate o-phenylenediamine dihydrochloride (Sigma) and read in an ELISA reader (Biotech) at 490 nm. The intensity of the signal correlates with the amount M1 produced during the infection. In all the steps, DMEM without serum contained 1 μg of L-1-tosylamide-2-phenylethyl chloromethyl ketone-treated trypsin (TPCK; Sigma) for activation of the virus infectivity. All tissue culture reagents were from GIBCO.

## Results and discussion

**Nlz** analogs, shown in Fig 2, have structural variations in the piperazine ring. Compounds **1** and **2** are analogs with the introduction of the 2,5-diazabicycle[2.2.1]heptane (DBH) system, where compound **1** corresponds to the (1*S*,4*S*) diastereomer and compound **2** corresponds to the (1*R*,4*R*) diastereomer. This system has been introduced in a variety of compounds [22] with antiparasitic [23], antibiotic [24], and anticarcinogenic activity [25]. It has been suggested that compounds with the DBH system have a better binding ability, compared to the piperazine analogs, due to the rigidity of the bicycle ring. The diamine system of 2,5-diazabicyclo[2.2.1]heptane is traditionally included in screening libraries as a rigid counterpart of the flexible piperazine ring [26–30]. Compounds **3** and **4** exemplify flexible ligands by substituting the piperazine ring for an ethylenediamine functional group. To assess the role of the nitrogen atoms in the piperazine, we analyzed compounds **5**, **6**, **7**, and **8**. These molecules allowed us to investigate the relevance of the distance among the nitrogen atoms of the piperazine by diminishing the number of carbon atoms between them.

### Molecular descriptors

Molecular descriptors can be informative of ADME profiles. Here we calculated descriptors with relevance in ADMET properties, with the software MOE 2022.10 [11] (see Supporting information). S1 Fig in S1 File shows a comparison of SlogP and logS values. The SlogP corresponds to the octanol/water partition coefficient, calculated for ~7000 structures [31], and is a measure of lipophilicity. logS is the log of the aqueous solubility (mol/L) calculated from a linear model. It is known that lipophilicity greatly impacts ADME properties due to its effect on solubility, plasma protein binding (PPB), metabolic clearance, the volume of distribution, and enzyme/receptor binding, among other pharmacological properties. Thus, as lipophilicity increases, solubility decreases, and the solubility increases as the logS increases (more positive).

S1 Fig in S1 File, shows the SlogP and logS for **nlz** and other commercial antivirals used for the treatment of the influenza infections, such as oseltamivir, zanamivir, amantadine, and rimantadine. Broad spectrum antivirals, such as arbidol and acyclovir, foscarnet, peramivir, remdesivir, nirmaltrevir or ritonavir, are also shown. To note, naproxen, acetaminophen, and chlorpheniramine are other drugs used in combination with antivirals to treat influenza infections.

The calculated values of SlogP for **nlz** and **Gerritz 3** are 4.17 and 4.18, respectively. These are higher values compared to those of commercial anti-flu drugs, amantadine, rimantadine, and oseltamivir; the difference between **nlz** and zanamivir is even more significant. The value of **nlz** is also greater than that of naproxen, acetaminophen, and chlorpheniramine. Not surprisingly, **nlz** and **Gerritz 3** have low aqueous solubility compared with rimantadine, amantadine, oseltamivir, and zanamivir. **Nlz** is also less soluble than naproxen, acetaminophen, and chlorpheniramine. Thus, although the high lipophilicity is the molecular characteristic that allowed the ligand to interact with the recognition site, this can also be undesirable in terms of pharmacokinetics and ADME properties due to low solubility. Nonetheless, arbidol and acyclovir, broad-spectrum antivirals, have higher lipophilicity than **nlz** and **Gerritz 3** and low solubility values.

S2 Fig in S1 File compares SlogP and logS for relevant modifications in **nlz** structure. S3 and S4 Figs in S1 File show the chemical structures for the analogs mentioned above. Modifications in isoxazole-4-caboxamide moiety have been reported in the literature. Derivatives of 1*H*-1,2,3-triazole-4-carboxamide are more soluble than **nlz**, even more than the 1*H*-pyrazol-4-carboxamides and phenyl-carboxamides derivatives. In an analog reported by Liao *et al*. [32], the absence of the isoxazol ring makes it less lipophilic and more aqueous soluble. In turn, changing the nitrogen atom of the aniline group for a methine group decreases the solubility and increases the lipophilicity [9].

S5 Fig in S1 File shows that compounds **1** and **2** are less soluble (logS = -6.57) than **nlz** (logS = -6.18) due to the introduction of the methylene bridge, compounds **3** and **4** were slightly more soluble (logS = -6.08 and -6.05, respectively). When the nitrogen atoms are removed in the piperazine moiety the value of logS diminished (logS = -6.61 for ligand **5** and -6.65 for compound **6**), the enolic tautomeric form of the compound **6** is more soluble than **nlz** (logS = -6.11) and for the case of the compound **7** are even less soluble with logS = -7.66 for the keto form and -7.12 for the enol form. Even with NH groups, compound **8** is less soluble (logS = -6.51) than **nlz**. Thus, the nitrogen atoms have an essential contribution to solubility.

### QSAR models

**Fig 6:**
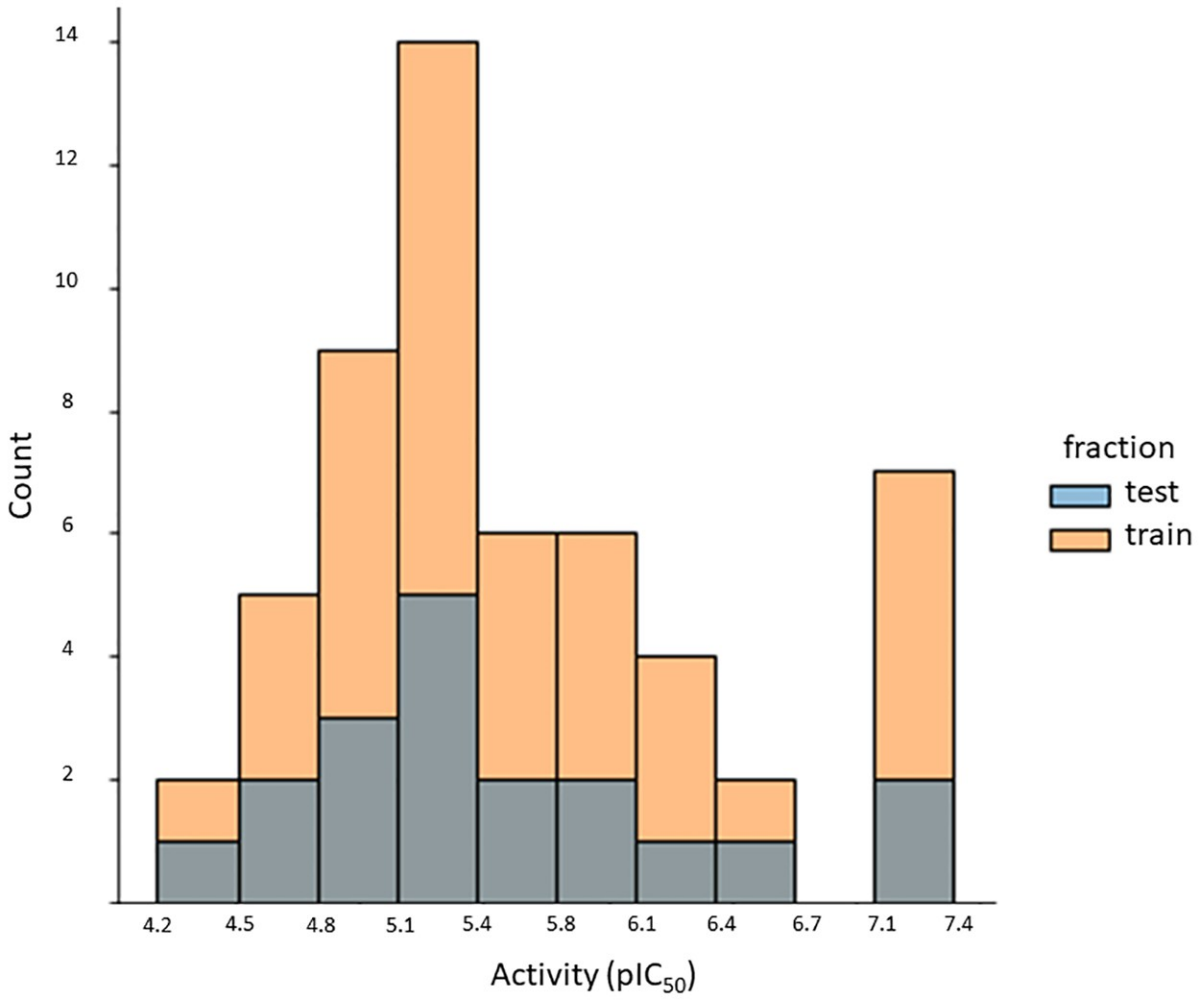
Distribution of pIC50 values for nucleozin derivatives. Molecules in the training set are shown in orange, and molecules in the test set are shown in gray. The inhibitory potency of the test set falls within the interval of pIC50 values of the training set.

The distribution of IC_50_ antiviral activity values of our curated dataset is shown in Fig 6. Activity values range from 4.2 to 7.4. The activity values of training and test sets lie within the same range.

In the preprocessing stage, features with low variance (zero or near to zero) were removed. Then, we transformed all the remaining features to have equal variance. By scaling the features, one gives an equal chance to all the features to influence the model. Thus, the standardized data can be informative. We employed StandardScaler as scaling algorithm. As a next step, we selected the most relevant features by assessing their importance using the meta-transformer SelectFromModel, alongside the estimators listed in S2 Table in S1 File. To limit the number of features, we first explored the best base estimator with the meta-transformer SelectFromModel; then, we fine-tuned this base estimator with the hyperparameters listed in S3 Table in S1 File. Lastly, the number of features was evaluated from 20 to 600 (S4 Table in S1 File), and the best models were obtained with 60 to 70 features.

The models were further refined with hyperparameter tuning [20]. The number of trees in the random forest varied from 50 to 5000. Above 600 trees, the statistics of the models were consistently improved (S3 Table in S1 File).

Max_depth, is the maximum depth of the tree. The default value is None, and then nodes are expanded until all leaves are pure or until all leaves contain less than min_samples_split samples (the hyperparameter min_samples_split is the minimum number of samples required to split an internal node, the default value is 2). Max_depth was used in an interval between 1 and 150 (S3 Table in S1 File). Here, the best statistics were reached at max_depth = 50.

The hyperparameter Min_samples_leaf is the minimum number of samples required at a leaf node. A split point at any depth will only be considered if it leaves at least min_samples_leaf training samples in each of the left and right branches. The default value is 1. In the model, Min_samples_leaf was tested in an interval between 2 and 20 (S3 Table in S1 File). Here, the best statistics were reached at Min_samples_leaf = 8.

The hyperparameter min_impurity_decrease refers to a float value, and the default value es equal to 0.0. A node will be split if this split induces a decrease of the impurity greater than or equal to this value. It took values between 1.0E^-15^ and 2.0 (S3 Table in S1 File). Here, the best statistics were reached at 1X10^-6^.

Lastly, warm_start is a hyperparameter with the Boolean value. The default is False; when set to True, reuse the solution of the previous call to fit and add more estimators to the ensemble; otherwise, just fit a whole new forest. Here, the best statistics were reached with True. All the other parameters were left as default.

The selected 60 or 70 features were further selected using PCA, reasonably good models contained 15 or more PC S5 Table in S1 File. After the preprocessing, models were generated with several regression algorithms, listed in Table 1, exploring from linear regression to Random Forest. Models with the best statistics were SVR and the variant NuSVR. Further refinement was performed using hyperparameter tuning.

**Table 1:** Search for the best regressor algorithm for training.

Model	Regressor	r^2^
1	LinearRegression	0.7706
2	Ridge	0.7823
3	Lasso	0.1625
4	ElasticNet	0.7556
5	BayesianRidge	0.7685
6	SGDRegressor	0.7542
7	KNearestNeighborRegressor	0.5791
8	SVR	0.9141
9	NuSVR	0.9103
10	LinearSVR	0.7275
11	DecisionTreeRegressor	1.0
12	RandomForestRegressor	0.9112
13	MLPRegressor	0.9405

Using SVR, the following step consisted of hyperparameter tuning (Table 2). The hyperparameters analyzed were C (the regularization parameter) and max_iter. The first one, C is a parameter that determines the strength of the regularization; it must be positive, and higher values of C correspond to less regularization. In these cases, the model tries to fit each data point of the training set as best as possible. At the same time, with low values of the parameter C, the algorithms put more emphasis on adjusting to the majority of data points [33]. The default value is 1.0. Here, the best statistics were reached at 2.0 (model 16 in Table 2). The hyperparameter max_iter is a hard limit on iterations within the solver; it is a positive integer value or -1 for no limit (default value). For the best model, max_iter = 100 000.

**Table 2:** Hyperparameter tuning for SVR regressor.

Model	Hyperparameter	r^2^
	*C*	
13	0.1	0.2713
14	0.5	0.7681
15	1.0 (default)	0.9039
16	2.0	0.9270
17	3.0	0.9259
18	4.0	0.9393
19	5.0	0.9458
20	10	0.9594
21	20	0.9698
22	50	0.9878
	*max_iter*	
31	-1	0.908255
32	100	0.891469
33	1000	0.894005
34	10000	0.883529
35	50000	0.89358
36	100000	0.90203

In summary, the best model was developed with SVR, with the hyperparameters C = 2.0 and max_iter = 100 000. The statistics obtained for that model are summarized in Table 3, and the code of Colab is available in the supporting information.

**Table 3:** Results for the support vector regressor.

	r^2^	q^2^_CV-LMO_ (Folds = 10)	q^2^_ext_
	0.94	0.60	0.60
MSE	0.03	0.25	0.21

**Fig 7:**
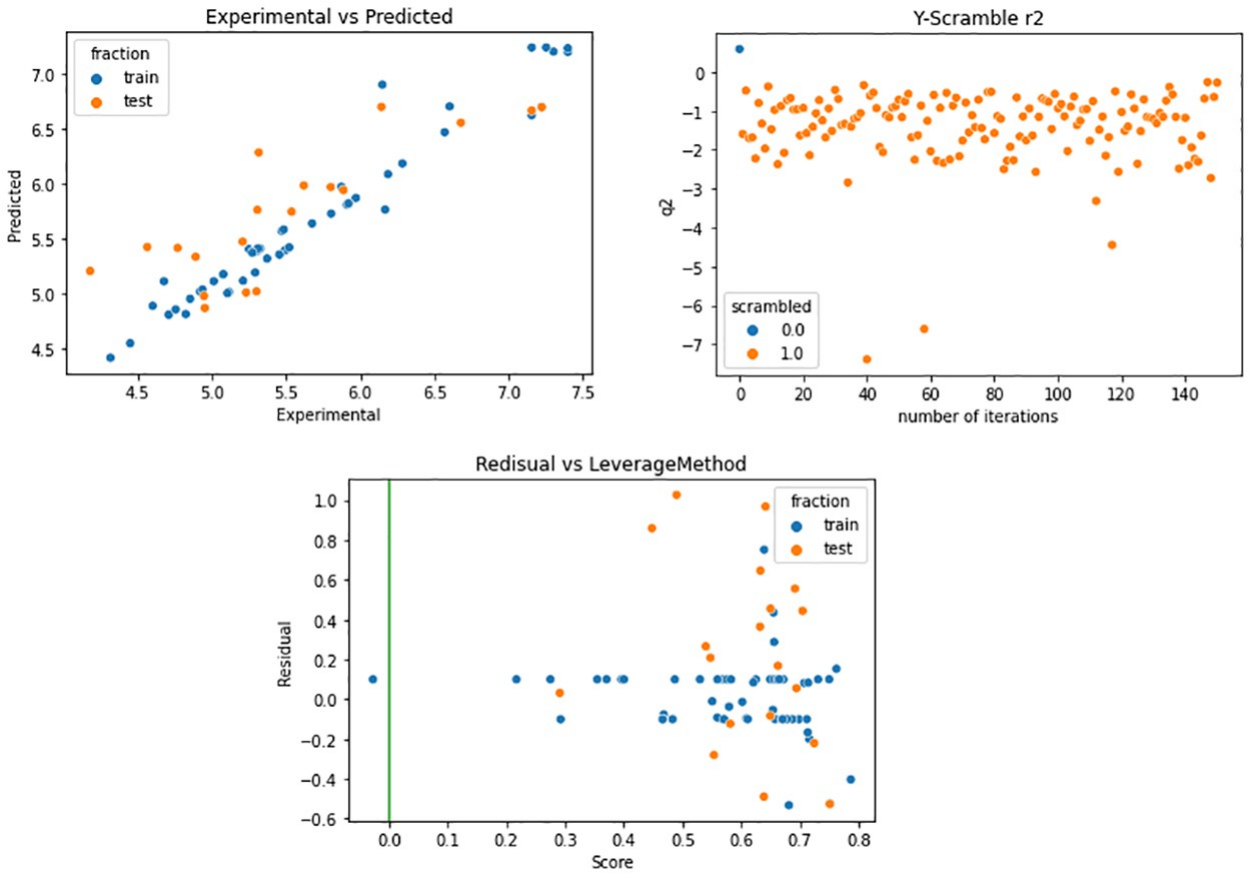
Results of QSAR model. **a)** Regression plot of the SVR model. The plot shows the predicted values by SVR model against the experimental for the train set (in blue) and test set (in orange). **b)** Y-Scramble test for the predicted model: In the plot is shown, in orange, the results of the q^2^_CV-LMO_ values for randomized models and in blue the q^2^_CV-LMO_ values for the true model (no randomized). **c)** Williams plot for the calculated model: The Applicability Domain was calculated by the leverage method.

Fig 7a shows the distribution plot of the values predicted with the SVR model and the experimental values, the train test is shown in blue, and the test set is shown in orange and is defined by a linear correlation. Fig 7b shows the results of the Y-scrambling test with 150 iterations (orange dots). As expected, the q^2^ obtained for the models generated after aleatorization decreases. They show that the model’s performance is not due to chance. Fig 7c shows the applicability domain using the Williams plot. Under that description, the model can define most of the molecules. The statistics of the best model are r^2^ = 0.94, q^2^ = 0.60, and q^2^_ext_ = 0.60. The activity predicted for analogs **1**–**12** is shown in Table 4. Interestingly, **Geritz 3** and **nlz** are predicted more actives than the **nlz** analogs, in agreement with the preliminary experimental data presented here.

**Table 4:** Predicted activity for nucleozin analogues.

Compound	Experimental	Predicted
Nlz	7.22	6.69
Gerritz 3	7.39	7.19
compound 1		5.87
compound 2		5.86
compound 3		6.03
compound 4		5.50
compound 5		6.60
compound 6		6.59
compound 6 enol		6.59
compound 7 anti		6.04
compound 7 enol		6.25
compound 7 syn		5.86
compound 8		5.34
compound 9		5.54
compound 10		5.29
compound 11		4.90
compound 12		5.06

### Synthesis

The reported synthetic procedure for nlz using 1,2-dichlorobenzen [2] was not reproducible in our lab. Thus, we designed a new method for the synthesis of **nlz**. The synthetic route is shown in Fig 3. Compound **13** was mixed with the secondary amine **14** in DMF at 120 °C, in the presence of K_2_CO_3_, to give compound **15** via a nucleophilic aromatic substitution reaction. The subsequent deprotection of the *N*-Boc group with 5.5 equivalents of methane sulfonic acid in dichloromethane yielded compound **16** quantitatively in five minutes. After this deprotection, a solution of amine **16** was added to a solution of a mixed anhydride, generated *in situ* from the carboxylic acid **17**. It was treated with pivaloyl chloride in the presence of triethylamine, as a base, in dry dichloromethane as the solvent; the consequent addition-elimination reaction gave the **nlz** compound.

The structures of **nlz** and analogues were characterized by ^1^H-NMR, ^13^C-NMR, DEPT-135, and the corresponding molecular weights were confirmed by DART mass spectrometry. The spectra and assignation of the signals are described in detail as Supporting Information.

The same synthetic strategy was employed for the synthesis of compounds **9** and **10** (Fig 4); for these compounds was used the amine (1*S*,4*S*)-*N*-Boc-2,5-diazabicycle[2.2.1]heptane, *N*-BocDBH, (**19**), reported previously, instead of the amine **14**. Compound **9** was prepared from 2-chloro-4-nitrofluorobenzene (**13**), and compound **10** from the compound 4-nitrofluorobenzene (**18**).

A similar methodology was also used to obtain compound **11**. The ethylenediamine was mono protected using the Boc_2_O reagent to obtain compound **24** which was mixed with an acyl chloride generated *in situ* from the carboxylic acid **17** treated with oxalyl chloride in dry dichloromethane in the presence of triethylamine as a base, and drops of dimethylformamide, rendering compound **25**. Then, for the deprotection of the *N*-Boc group, the compound **25** was treated with methane sulfonic acid to form the primary amine **26**, followed by a nucleophilic aromatic substitution, using **13** as an electrophile for obtaining the compound **11** (Fig 5).

Thus, here we employed a new route using 4-nitrofluroarenes as feedstock, which efficiently led to **nlz** and some derivatives. The aromatic nucleophilic substitution of the fluor atom was performed in dimethylformamide (DMF) as solvent at 120 °C. Some observations in the laboratory indicated that it was not necessary to add DMF to the reaction mixture for the synthesis of compound **20**. For this reason, we carried out the reaction grinding in a mortar with a pestle, for 15 minutes, the monoprotected diamine **19** and the compound **13**, both in solid state, in the presence of five equivalents of K_2_CO_3_ as a base, without DMF. The reaction was monitored by thin-layer chromatography (TLC), comparing the reaction mixture against the previously characterized compound **20**, showing that most of the raw materials were transformed. Compound **20** was obtained by the addition of 3 mL of hot ethyl acetate (AcOEt), filtered through a small layer of Celite, recrystallized, and isolated from the mother liquor. By thin layer chromatography, a product with a good degree of purity was observed, and the melting point coincided with that reported m.p. = 199.7 °C. Thus, the substitution reaction can be carried out under free-solvent conditions. Future experiments will be carried out to explore this methodology as a potential synthesis route to new **nlz** derivatives.

#### Biological evaluation

**Fig 8:**
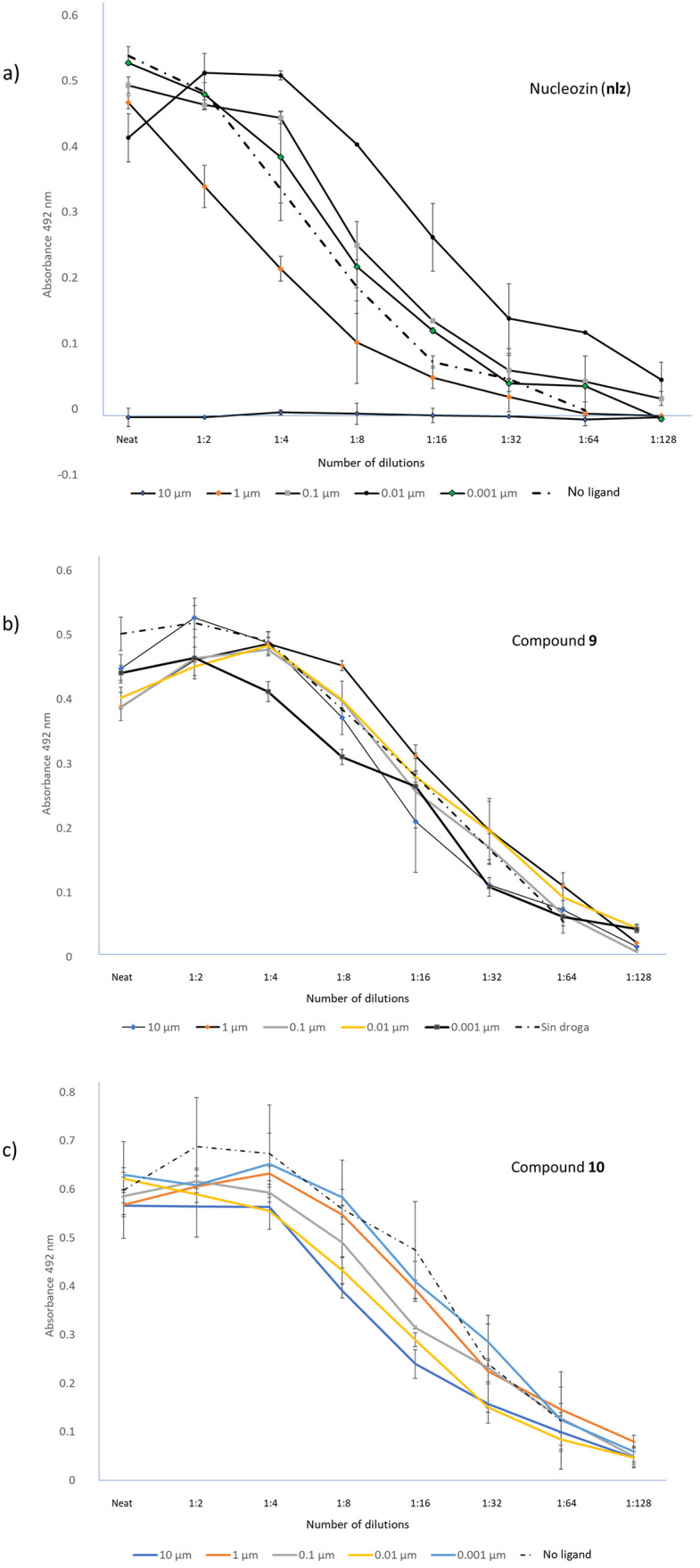
Biological evaluation of nlz and analogs in cell cultures of influenza virus-infected MDCK cells. The experiments were performed by infecting MDCK cells with the Influenza virus A/Caledonia H1N1, at a concentration of 0.001 pfu per cell in presence of various concentrations of **nlz** or analogs. After 48 hrs of incubation, the supernatants were obtained and titrated in MDCK cells and incubated for 72 hrs. Cells were fixed-permeabilized and an ELISA on the cells performed, using an anti- influenza M1 monoclonal antibody, followed by a goat anti-mouse Ig’s polyclonal antibody conjugated to HRP. After adding the substrate, the color intensity was read at 492 nm in an automated ELISA plate reader. **a**) Evaluation of **nlz**. **b**) biological evaluation of the ligand **9**. **c**) biological evaluation of the ligand **10**.

The virus load starts to decline in the presence of 1μM of **nlz** and is totally inhibited at 10 μM. Under the same conditions but in the presence of analogs **9** and **10**, viral growth was not inhibited, as is shown in Fig 8. It is possible that analogs **9** and **10** will have viral inhibitory effects at larger concentrations. These results show that constraining the core structure of **nlz** (analogs **9** and **10**) results in the loss of activity. Interestingly, the QSAR model developed here predicts the analogs **9** and **10** as less active, consistent with the experimental observation.

Additional molecular modeling studies and experimental evaluation of other analogs will provide information on structural modifications required for the improvement of antiviral activity. QSAR models developed here will aid the synthetic efforts of new **nlz** analogs.

## Supporting information

S1 File Inhibitory activity data of the influenza A/WSN/33 (H1N1) virus, computational details, and synthetic details. (PDF)
