# Supplementary material for: Modifications in the piperazine ring of nucleozin affect anti-influenza activity
Source: PLoS One. 2023 Feb 10;18(2):e0277073. doi: 10.1371/journal.pone.0277073 (PMC9916566; doi:10.1371/journal.pone.0277073)
Supplement: S1 File — (PDF) [file pone.0277073.s001.pdf]

Modifications in the piperazine ring of nucleozin affect anti-influenza activity

Erick Correa-Padilla[1,2], Alejandro Hernández-Cano[1,3], Gabriel Cuevas[1], Yunuen Acevedo-Betancur[4], Fernando Esquivel-Guadarrama[4], and Karina Martinez-Mayorga[1,5,6,*]

[1] Institute of Chemistry, National Autonomous University of Mexico, Mexico City, Mexico.
[2] Zaragoza School of Higher Education, National Autonomous University of Mexico, Mexico City, Mexico
[3] School of Science, National Autonomous University of Mexico, Mexico City, Mexico.
[4] School of Medicine, Autonomous University of the State of Morelos, 62100 Cuernavaca, Morelos, México.
[5] Institute of Chemistry, Campus Merida, National Autonomous University of Mexico, Merida-Tetiz Highway, Km. 4.5, Ucú, Yucatán, México.
[6] Institute for Applied Mathematics, Merida Research Unit, National Autonomous University of Mexico, Sierra Papacal Merida, Yucatan, Mexico.

Supporting information

S1 Table. Inhibitory activity data of the influenza A/WSN/33 (H1N1) virus with the McIP assay, reported by Cheng et al.

Compound	IC_{50} (μM)	pIC_{50}	SMILES
Cheng 2a	4.83	5.316052869	Clc1cc([N+](=O)[O-])ccc1N1CCN(CC1)C(=O)c1c(onc1C)-c1ccccc1
Cheng 2b	0.71	6.148741651	Clc1cc([N+](=O)[O-])ccc1N1CCN(CC1)C(=O)c1c(onc1C)-c1ccccc1OC
Cheng 3a	3.13	5.504455662	Clc1cc([N+](=O)[O-])ccc1N1CCN(CC1)C(=O)c1n(nnc1C)-c1ccccc1
Cheng 3b	0.68	6.167491087	Clc1cc([N+](=O)[O-])ccc1N1CCN(CC1)C(=O)c1n(nnc1C)-c1ccccc1OC
Cheng 3c	5.35	5.271646218	Clc1cc([N+](=O)[O-])ccc1N1CCN(CC1)C(=O)c1n(nnc1C)-c1cc(OC)ccc1
Cheng 3d	5.61	5.251037139	Clc1cc([N+](=O)[O-])ccc1N1CCN(CC1)C(=O)c1n(nnc1C)-c1ccc(OC)cc1
Cheng 3e	1.2	5.920818754	Clc1cc([N+](=O)[O-])ccc1N1CCN(CC1)C(=O)c1n(nnc1C)-c1ccccc1Cl
Cheng 3f	4.89	5.310691141	Clc1cc([N+](=O)[O-])ccc1N1CCN(CC1)C(=O)c1n(nnc1C)-c1cc(Cl)ccc1
Cheng 3g	4.93	5.307153081	Clc1cc([N+](=O)[O-])ccc1N1CCN(CC1)C(=O)c1n(nnc1C)-c1ccc(Cl)cc1
Cheng 3h	1.58	5.801342913	Clc1cc([N+](=O)[O-])ccc1N1CCN(CC1)C(=O)c1n(nnc1C)-c1ccccc1O
Cheng 3i	2.4	5.619788758	Clc1cc([N+](=O)[O-])ccc1N1CCN(CC1)C(=O)c1n(nnc1C)-c1ccccc1C
Cheng 3j	3.22	5.492144128	Clc1cc([N+](=O)[O-])ccc1N1CCN(CC1)C(=O)c1n(nnc1C)-c1ccccc1OCC
Cheng 3k	8.42	5.074687909	Clc1cc([N+](=O)[O-])ccc1N1CCN(CC1)C(=O)c1n(nnc1C)-c1ccccc1OC(C)C

Cheng 3m	1.34	5.872895202	Clc1cc([N+](=O)[O-])ccc1N1CCN(CC1)C(=O)c1n(nnc1C)-c1c(OC)cccc1OC
Cheng 3n	8.36	5.077793723	Clc1cc([N+](O)=O)ccc1N1CCN(CC1)C(=O)c1n(nnc1C)-c1ccccc1OC
Cheng 3p	48.12	4.317674381	Clc1ccccc1N1CCN(CC1)C(=O)c1n(nnc1C)-c1ccccc1OC
Cheng 3q	16.98	4.770062314	O(C)c1ccccc1-n1nnc(C)c1C(=O)N1CCN(CC1)c1ccc([N+](=O)[O-])cc1
Cheng 3t	35.45	4.45038376	Clc1cc([N+](=O)[O-])ccc1N1CCCN(CC1)C(=O)c1n(nnc1C)-c1ccccc1OC
Cheng 4a	3.38	5.4710833	Clc1cc([N+](=O)[O-])ccc1N1CCN(CC1)C(=O)c1c(n[nH]c1C)-c1ccccc1
Cheng 4b	0.72	6.142667504	Clc1ccccc1-c1n[nH]c(C)c1C(=O)N1CCN(CC1)c1ccc([N+](=O)[O-])cc1Cl
Cheng 5a	5.24	5.280668713	Clc1cc([N+](=O)[O-])ccc1N1CCN(CC1)C(=O)c1ccccc1-c1ccccc1
Cheng 5b	1.57	5.804100348	Clc1cc([N+](=O)[O-])ccc1N1CCN(CC1)C(=O)c1ccccc1-c1ccccc1OC
Gerritz 3	0.04	7.397940009	Clc1cc([N+](=O)[O-])ccc1N1CCN(CC1)C(=O)c1c(noc1C)-c1ccccc1OC
Gerritz 4	0.07	7.15490196	Clc1cc([N+](=O)[O-])ccc1N1CCN(CC1)C(=O)c1c(noc1C)-c1cccnc1Cl
Gerritz 5	0.07	7.15490196	Clc1cc([N+](=O)[O-])ccc1N1CCN(CC1)C(=O)c1n(nnc1-c1ccccc1OC)C
Kao 1	0.05	7.301029996	Clc1cc([N+](=O)[O-])ccc1N1CCN(CC1)C(=O)c1c(noc1C)-c1ccc(O)cc1
Kao 10	25	4.602059991	Clc1ccccc1-c1noc(C)c1C(=O)N1CCN(CC1)c1c([N+](=O)[O-])cccc1Cl
Kao 2	0.06	7.22184875	Clc1cc([N+](=O)[O-])ccc1N1CCN(CC1)C(=O)c1c(noc1C)-c1ccccc1
Kao 3	0.056	7.251811973	Clc1cc([N+](=O)[O-])ccc1N1CCN(CC1)C(=O)c1c(noc1C)-c1ccc(N)cc1
Kao 4	0.25	6.602059991	Clc1cc([N+](=O)[O-])ccc1N1CCN(CC1)C(=O)c1c(noc1C)-c1ccc(N=[N+]=[N-])cc1
Kao 5	0.04	7.397940009	Clc1ccccc1-c1noc(C)c1C(=O)N1CCN(CC1)c1ccc([N+](=O)[O-])cc1Cl
Kao 6 (R)	0.21	6.677780705	Clc1ccccc1-c1noc(C)c1C(=O)N1CCN(CC1C)c1ccc([N+](=O)[O-])cc1Cl
Kao 8	5.1	5.292429824	Clc1ccccc1-c1noc(C)c1C(=O)N1CCN(CC1)c1ccc([N+](=O)[O-])cc1
Kao 9	12	4.920818754	Clc1cccc(Cl)c1-c1noc(C)c1C(=O)N1CCN(CC1)c1ccc([N+](=O)[O-])cc1
Liao 10	19.52	4.709520187	Clc1cc([N+](=O)[O-])ccc1N1CCN(CC1)C(=O)c1ccccc1
Liao 11	5.2	5.283996656	Clc1cc([N+](=O)[O-])ccc1N1CCN(CC1)C(=O)c1ccccc1C
Liao 12	14.96	4.825068406	Clc1cc([N+](=O)[O-])ccc1N1CCN(CC1)C(=O)c1cc(ccc1)C

Liao 13	5.87	5.231361899	Clc1cc([N+](=O)[O-])ccc1N1CCN(CC1)C(=O)c1ccc(cc1)C
Liao 14	4.93	5.307153081	Clc1ccccc1C(=O)N1CCN(CC1)c1ccc([N+](=O)[O-])cc1Cl
Liao 15	11.14	4.953114809	Clc1cc([N+](=O)[O-])ccc1N1CCN(CC1)C(=O)c1cc(Cl)ccc1
Liao 16	7.66	5.11577123	Clc1cc([N+](=O)[O-])ccc1N1CCN(CC1)C(=O)c1ccc(Cl)cc1
Liao 17	4.24	5.372634143	Brc1ccccc1C(=O)N1CCN(CC1)c1ccc([N+](=O)[O-])cc1Cl
Liao 18	11.28	4.9476909	Brc1cc(ccc1)C(=O)N1CCN(CC1)c1ccc([N+](=O)[O-])cc1Cl
Liao 19	7.85	5.105130343	Brc1ccc(cc1)C(=O)N1CCN(CC1)c1ccc([N+](=O)[O-])cc1Cl
Liao 20	6.2	5.207608311	Clc1cc([N+](=O)[O-])ccc1N1CCN(CC1)C(=O)c1ccccc1[N+](=O)[O-]
Liao 21	27.19	4.565590792	Clc1cc([N+](=O)[O-])ccc1N1CCN(CC1)C(=O)c1cc([N+](=O)[O-])ccc1
Liao 22	3.01	5.521433504	Clc1cc([N+](=O)[O-])ccc1N1CCN(CC1)C(=O)c1ccc([N+](=O)[O-])cc1
Liao 23	4.72	5.326058001	Clc1cc([N+](=O)[O-])ccc1N1CCN(CC1)C(=O)c1ccccc1O
Liao 24	12.84	4.891434976	Clc1cc([N+](=O)[O-])ccc1N1CCN(CC1)C(=O)c1ccccc1N
Liao 25	9.69	5.013676223	Clc1cc([N+](=O)[O-])ccc1N1CCN(CC1)C(=O)c1ccccc1CC
Liao 26	11.55	4.937418016	Clc1cc([N+](=O)[O-])ccc1N1CCN(CC1)C(=O)c1ccccc1C1CCCCC1
Liao 27	6.16	5.210419288	Clc1cc([N+](=O)[O-])ccc1N1CCN(CC1)C(=O)c1ccccc1OC
Liao 28	3.5	5.455931956	Clc1cc([N+](=O)[O-])ccc1N1CCN(CC1)C(=O)c1ccc(cc1)C#N
Liao 29	66.03	4.180258703	Clc1cc([N+](=O)[O-])ccc1N1CCN(CC1)C(=O)c1ccc(cc1)C(OC)=O
Liao 30	0.65	6.187086643	Clc1cc([N+](=O)[O-])ccc1N1CCN(CC1)C(=O)c1c(cccc1N)C
Liao 31	3.28	5.484126156	Clc1cc([N+](=O)[O-])ccc1N1CCN(CC1)C(=O)c1c(cccc1[N+](=O)[O-])C
Liao 32	1.07	5.970616222	Brc1cccc(C)c1C(=O)N1CCN(CC1)c1ccc([N+](=O)[O-])cc1Cl
Liao 33	0.52	6.283996656	Clc1cccc(C)c1C(=O)N1CCN(CC1)c1ccc([N+](=O)[O-])cc1Cl
Liao 34	4.88	5.311580178	Clc1cc([N+](=O)[O-])ccc1N1CCN(CC1)C(=O)c1c(cccc1C)C
Liao 35	2.11	5.675717545	Clc1cc([N+](=O)[O-])ccc1N1CCN(CC1)C(=O)c1ccc([N+](=O)[O-])cc1C
Liao 36	2.9	5.537602002	Brc1cc(C)c(cc1)C(=O)N1CCN(CC1)c1ccc([N+](=O)[O-])cc1Cl

Liao 37	1.23	5.910094889	Brc1cc([N+](=O)[O-])ccc1C(=O)N1CCN(CC1)c1ccc([N+](=O)[O-])cc1Cl
Liao 38	5.33	5.273272791	Brc1cc(F)c(cc1)C(=O)N1CCN(CC1)c1ccc([N+](=O)[O-])cc1Cl
Lia0 39	0.27	6.568636236	Clc1cc(cc(C)c1C(=O)N1CCN(CC1)c1ccc([N+](=O)[O-])cc1Cl)C#N
Nucleozin	0.06	7.22184875	Clc1cc([N+](=O)[O-])ccc1N1CCN(CC1)C(=O)c1c(noc1C)-c1ccccc1
Su 1 (3061)	0.07	7.15490196	Clc1ccccc1-c1noc(C)c1C(=O)N1CCN(CC1)c1ccc([N+](=O)[O-])cc1Cl
Su 10 9168	19.5	4.709965389	Clc1cccc([N+](=O)[O-])c1N1CCN(CC1)C(=O)c1c(noc1C)-c1ccccc1
Su 2 4332	1.3	5.886056648	Clc1cc([N+](=O)[O-])ccc1N1CC(N(CC1)C(=O)c1c(noc1C)-c1ccccc1)C
Su 3 2130	3.5	5.455931956	Clc1ccccc1-c1noc(C)c1C(=O)N1CCN(CC1)c1ccc(Cl)cc1[N+](=O)[O-]
Su 4 (3822)	1.2	5.920818754	o1nc(-c2ccccc2OC)c(C(=O)N2CCN(CC2)c2ccc([N+](=O)[O-])cc2)c1C
Su 5 6074	5	5.301029996	Clc1cccc(Cl)c1-c1noc(C)c1C(=O)N1CCN(CC1)c1ccc([N+](=O)[O-])cc1
Su 6 0927	14	4.853871964	Clc1ccccc1-c1noc(C)c1C(=O)N1CCN(CC1)c1c([N+](=O)[O-])cccc1Cl
Su 7 0131	17.5	4.756961951	Clc1ccccc1-c1noc(C)c1C(=O)N1CCN(CC1)c1cc(N2CCCC2)c([N+](=O)[O-])cc1
Su 9 4427	21	4.677780705	Clc1ccccc1-c1noc(C)c1C(=O)N1CCN(CC1)c1ccc([N+](=O)[O-])cc1

S2 Table. Evaluation of base estimators.

Model	Base estimator	r^2	q^2_{cv}
1	RandomForest	0.4568	0.4047
2	LinearRegression	0.6625	0.3058
3	Ridge	0.6773	0.3097
4	Lasso	---	---
5	ElasticNet	0.9248	----
6	ElasticNetCV	0.941	0.2496
7	BayesianRidge	0.6648	0.3083
8	SGDRegressor	0.9241	0.0657
9	KNearestNeighborRegressor	---	----
10	DesicionTree	0.9543	----
11	MLPregressor	---	----

S3 Table. Hyperparameter tuning for RandomForest regressor in the SelectFromModel algorithm.

Model	Hyperparameter	r^2
	Number of trees	
1	50	0.889726
2	100	0.889627
3	200	0.892266
4	300	0.911922
5	400	0.884414
6	500	0.908299
7	600	0.894403
8	700	0.895586
9	800	0.907335
10	900	0.896408
11	1000	0.907692
12	1500	0.47191
13	2000	0.906627
14	3000	0.903815
15	5000	0.889724
	max_depth	
19	1	0.914554
20	5	0.899148
21	10	0.906564
22	20	0.907206
23	50	0.90605
24	60	0.89697
25	70	0.90496
26	100	0.90612
27	150	0.8876
	min_samples_leaf	

43	2	0.905306
44	3	0.897723
45	4	0.909282
46	5	0.90568
47	6	0.908785
48	7	0.909276
49	8	0.899425
50	9	0.911808
51	10	0.902544
52	11	0.910523
53	15	0.859015
54	20	0.679566
	min_impurity_decrease	
80	1.0E-15	0.896716
81	1.0E-12	0.880198
82	1.0E-9	0.891953
83	1.0E-6	0.891708
84	1.0 E-3	0.904586
85	1.0 E-2	0.895698
86	1.0 E-1	0.917172
87	1.0	0.679566
88	2.0	0.679566
	warm_start	
89	False (default)	0.911189
90	True	0.891056

S4 Table. Search of the maximum number of features.

Model	max features	r^2	q^2_{cv}
1	257 (default)	0.907362	0.475546
2	600	0.907362	0.475546
3	300	0.907362	0.475546
4	250	0.907142	0.475546
5	200	0.916146	0.471066
6	150	0.909878	0.483206
7	100	0.915111	0.509704
8	90	0.91515	0.513279
9	80	0.917171	0.499364
10	70	0.918393	0.494183
11	60	0.918495	0.51263
12	50	0.912338	0.493
13	40	0.901462	0.377326
14	30	0.913563	0.370986
15	20	0.900508	0.357616

S5 Table. Search of relevant Principal Components.

Model	#PCA	r^2	q^2_{cv}
1	40	0.9386	0.4863
2	30	0.9276	0.4823
3	20	0.9073	0.4755
4	19	0.9055	0.4766
5	18	0.9036	0.4643
6	17	0.9026	0.4658
7	16	0.8975	0.4558
8	15	0.8950	0.4497
9	14	0.8914	0.4333
10	13	0.8919	0.4285
11	12	0.8919	0.4213
12	11	0.8856	0.4160
13	10	0.8609	0.3741
14	9	0.8018	0.3236
15	5	0.6663	0.1834

Experimental procedure

Reagents and chemicals: The chemicals and solvents were purchased from Sigma-Aldrich or from other commercial suppliers. The chemicals were used without further purification and the solvents were distillated prior use. Melting points were determined in open capillaries in a KRUSS melting points apparatus and in a Fischer-Jones melting point apparatus and are uncorrected. Reaction progresses were monitored by thin layer chromatography (TLC) using silica gel 60 F254 plates (Merck, A.G. Germany). Shortwave UV light (254 nm) and/or staining with iodine and ninhydrin solution, were used to monitor the reactions. Flash chromatography on silica gel technical grade (Merck, 230-400 mesh) was used to purify compounds and intermediates. ^{1}H and ^{13}C-NMR spectra were obtained either in a Bruker Avance 300 MHz, a Bruker Avance III 400 MHz, or a Bruker Ascend 500 MHz, using $CDCl_3$ or DMSO-d6 as solvents. Data for ^{1}H-NMR is referenced to the solvent ($CDCl_3$ = δ 7.26 for ^{1}H, δ 77.1 for ^{13}C; DMSO-d6 = δ 3.3 for ^{1}H (residual H_2O), δ 40.14 for ^{13}C). Low-resolution DART+ mass spectra were obtained on a Jeol JMS-T100LC spectrometer. Low-resolution Electronic Impact mass spectrum was obtained on a Jeol, SX 102 A spectrometer and values of the signals are expressed in mass/charge units (m/z), followed by the relative intensity with reference to a 100% base peak.

Synthesis of compounds 15, 20 and 21.

In a 25 mL round-bottom flask were mixed (4.69 mmol) of *N*-Boc monoprotected amine (**14** and **19**) with 4.69 mmol of the corresponding 4-nitro-fluorophenyl compound, and 1.5 g of K_2CO_3 (10.85 mmol) and 10 mL of DMF; the mixture was heated at 120 °C with magnetic stirring overnight and the end of the reaction was confirmed by TLC. DMF was eliminated on a rotary evaporator, and then 15 mL of DCM were added, the organic phase was washed twice with 20 mL of water and once with 20 mL of brine, dried over anhydrous Na_2SO_4, filtered and evaporated to dryness. It was recrystallized from 20 parts of AcOEt.

Solid-state synthesis of compound 20.

The monoprotected diamine **19** (50mg, 0.025 mmol) and the compound **13** (44mg, 0.25mmol), both in solid state, were grinded in a mortar with a pestle for 15 minutes, with five equivalents of K_2CO_3 (172mg, 1.25 mmol) as base. The reaction was monitored by TLC, 3 mL of hot AcOEt were added to the reaction medium, stirred for a minute, filtered through celite and recrystallized. The mother liquor was removed through decantation and then by suction with a Pasteur pipette, 11 mg of a yellow solid was obtained with a melting point of 200°C.

Compound **20:** m.p. 199.7 °C; **^{1}H-NMR** (500 MHz, $CDCl_3$): δ 8.17 (d, *J* = 2.0 Hz, 1H), 7.98 (d, *J* = 9.0 Hz, 1H), 6.64 (d, *J* = 8.8 Hz, 1H), 4.78 (d, *J* = 40.79 Hz, 1H), 4.59 (d, *J* = 69.61 Hz, 1H), 4.01 (dd, *J* = 23.0, 9.2 Hz, 1H), 3.61 (dd, *J* = 44.0, 9.9 Hz, 1H), 3.45 (m, 2H), 1.99 (d, *J* = 11.5 Hz, 2H), 1.44 (d, *J* = 12.2 Hz, 9H). **^{13}C-NMR** (125 MHz, $CDCl_3$): δ 154.13 (d, 5.22 Hz), 149.54, 138.50 (d, *J* = 13.71 Hz), 128.37, 123.72, 118.61 (d, *J* = 12.89 Hz), 114.33 (d, *J* = 27.29 Hz), 80.15 (d, *J* = 11.54 Hz), 60.39 (d, *J* = 72.59 Hz, CH), 59.85 (d, *J* = 41.37 Hz, CH_2), 56.52 (d, *J* = 130.03 Hz, CH), 52.19 (d, *J* = 85.19 Hz, CH_2), 37.13 (d, *J* = 45.38 Hz, CH_2), 28.44. **EI-MS** (70eV) m/z (% intensity): 353 (M^+,10), 337 (<5), 280 (22), 252 (20), 252 (18), 223 (56), 183 (12).

Compound **21:** m.p. 200.3 °C; **^{1}H-NMR** (500 MHz, $CDCl_3$): δ 8.11 (d, *J* = 8.8 Hz, 2H), 6.49 (d, *J* = 8.8 Hz, 2H), 4.64 (d, *J* = 65.8 Hz, 1H), 4.53 (s, 1H), 3.59 (d, *J* = 8.8 Hz, 1H), 3.49 – 3.24 (m, 3H), 2.00 (m, 2H), 1.44 (d, *J* = 24.2 Hz, 9H). **^{13}C-NMR** (125 MHz, $CDCl_3$): δ 152.97, 150.03, 136.42, 125.38 (2C), 109.64 (2C), 79.12 (d, *J* = 10.0 Hz), 56.60 (d, *J* = 63.41 Hz, CH), 55.71 (d, *J* = 27.93 Hz, CH_2), 55.33 (d, *J* = 118.9 Hz, CH), 51.06 (d, *J* = 50.43 Hz, CH_2), 36.47(d, *J* = 58.28 Hz, CH_2), 27.37 (*J* = 10.50 Hz).

Synthesis of compounds 16, 22, 22 and 25.

In a 50 mL round-bottom flask, 2.3 mmol of the monoprotected *N*-Boc-*N´*-(4-nitroaryl) amine (compounds **15**, **20**, **21**) were dissolved in 10 mL of DCM, and then 840 µL of $MeSO_3H$ were added slowly. The reaction, monitored by TLC, was completed in 5 minutes. Saturated Na_2CO_3 solution was added to pH > 8, extracted with DCM (2 X 10 mL), dried over anhydrous Na_2SO_4 and evaporated to dryness.

Compound **22:** m.p. 154.5 °C; **RMN ^{1}H** (500 MHz, $CDCl_3$) δ 8.19 (d, *J* = 2.5 Hz, 1H), 7.98 (dd, *J* = 9.3, 2.5 Hz, 1H), 6.63 (d, *J* = 9.3 Hz, 1H), 4.66 (s, 1H), 4.09 (dd, *J* = 9.7, 1.6 Hz, 1H), 3.83 (s, 1H), 3.35 – 3.21 (m, 2H), 3.16 (d, *J* = 10.1 Hz, 1H), 1.96 (d, *J* = 9.9 Hz, 1H), 1.89 (d, *J* = 9.9 Hz, 1H), 1.55 (s, 1H, NH). **RMN ^{13}C** (125 MHz, $CDCl_3$) δ 148.62 (0.5 C), 144.92 (0.5 C), 142.81 (0.5 C),137.08 (0.5 C), 127.36, 122.62, 117.52, 113.27, 61.64 (CH_2), 59.25 (CH), 55.27 (CH), 49.74 (CH_2), 36.16 (CH_2).

Compound **23:** m.p. 150.4 °C **^{1}H-NMR** (500 MHz, $CDCl_3$) δ 8.06 (d, *J* = 9.1 Hz, 2H), 6.44 (d, *J* = 9.0 Hz, 2H), 4.43 (s, 1H), 3.87 (s, 1H), 3.62 (d, *J* = 9.16 Hz, 1H), 3.11 (t, *J* = 9.56 Hz, 2H), 3.03 (d, *J* = 9.9 Hz, 1H), 1.94 (d, *J* = 9.80 Hz, 1H), 1.88 (d, *J* = 9.8 Hz, 1H), 1.59 (br, 1H). **RMN ^{13}C** (125 MHz, $CDCl_3$) δ 146.47, 132.06, 121.61 (2C), 105.76 (2C), 54.59 (CH_2), 52.90 (CH), 51.38 (CH), 46.12 (CH_2), 32.85 (CH_2).

Synthesis of nlz, 9 and 10.

In a 25 mL ball flask, 240 mg (1.2 mmol) of compound **17** were placed with 5 mL of dry DCM and 350 mL of TEA were added, cooled between 0 and 5 ºC, 154 mL of PivCl were added and stirred for 30 min. It was confirmed by TLC that all the carboxylic acid **17** reacted and then 1.2 mmol of the corresponding amine was added in solid state and stirred for 30 minutes. Then, 5 mL of water were added and stirred for a few minutes, the phases were separated and extracted with 5 mL of DCM, the organic phase was washed with 10 mL of water, dried over anhydrous Na_2SO_4, evaporated to dryness, and recrystallized from ethyl acetate, obtaining 1.3 g of a yellow solid with a melting point of 174.5 °C, 25% yield.

Nucleozin (nlz): m.p. 174.5 °C; **^{1}H-RMN** (300 MHz, $CDCl_3$) δ 8.21 (d, *J* = 2.6 Hz, 1H), 8.05 (dd, *J* = 8.9, 2.6 Hz, 1H), 7.70 – 7.62 (m, 2H), 7.52 – 7.45 (m, 3H), 6.84 (d, *J* = 8.9 Hz, 1H), 3.92 (m, 2H), 3.28 (m, 2H), 3.12 (m, 2H), 2.54 (m, 5H). **^{13}C-NMR** (75 MHz, $CDCl_3$) δ 169.98, 162.56, 159.96, 153.90, 142.79, 130.38, 129.10 (2C), 128.36, 128.00, 127.86 (2C), 126.54, 123.38, 119.55, 110.94, 50.14 (2C), 46.87, 41.85, 11.83. **LRMS** (DART+) m/z (% intensity): 429 (35, M^++2), 427 (100, M^+), 226 (17).

Compound **9:** m.p. 174.5 °C; **^{1}H-NMR** (500 MHz, $CDCl_3$) δ 8.14 (dd, *J* = 17.7, 2.3 Hz, 1H), 7.90 (ddd, *J* = 72.9, 9.2, 2.2 Hz, 1H), 7.63-7.06 (m, 5H), 6.42 (dd, *J* = 130.4, 9.2 Hz, 1H), 5.15 (s, 1H), 4.63 (d, *J* = 86.1 Hz, 1H), 4.07 (m,1H),

3.82 - 3.42 (m, 2H), 3.11 (dd, *J* = 39.3, 9.8 Hz, 1H), 2.53 (d, *J* = 19.9 Hz, 3H), 1.95 (dd, *J* = 89.13, 10.23 Hz, 1H), 1.70 (dd, *J* = 108.57, 9.98 Hz, 1H). **^{13}C-NMR** (125 MHz, $CDCl_3$) δ 170.04 (d, *J* = 42.24 Hz), 161.57 (d, *J* = 88.36 Hz), 159.67 (d, *J* = 38.84 Hz), 148.97 (d, *J* = 24.56 Hz), 138.92 (d, *J* = 29.21 Hz), 130.30 (d, J = 67.75 Hz), 128.91 (d, *J* = 56.21 Hz), 128.22 (d, *J* = 13.37 Hz, 2C), 127.92 (d, *J* = 51.08 Hz), 126.83 (2C), 123.54 (d, *J* = 7.65 Hz), 118.93 (d, *J* = 58.13 Hz), 114.23 (d, *J* = 67.58 Hz), 111.65 (d, *J* = 46.35 Hz), 59.49 (m, CH), 59.01 (d, *J* = 20.47 Hz, CH_2), 55.63, 52.29 (d, *J* = 190.98 Hz, CH_2), 36.77 (d, *J* = 137.43 Hz, CH_2), 11.81. **LRMS** (DART+) m/z (% intensity): 441 (35, M^++2), 439 (100, M^+).

Compound **10:** m.p. 196.2 °C; **^{1}H-NMR** (500 MHz, $CDCl_3$): δ 7.98 (dd, *J* = 23.7, 9.0 Hz, 1H), 7.56(d, *J* = 7.56 Hz, 1H) 7.49 – 7.27 (m, 3H), 7.10 (dt, *J* = 38.5, 7.4 Hz, 2H), 6.21 (d, *J* = 8.9 Hz, 1H), 5.11 (s, 0.5H), 4.48 (s, 0.5H), 4.26 (s, 0.5 H), 4.01 (s, 0.5 H) – 3.90 (m, 1H), 3.63 – 2.05 (m, 4H), 2.43 (d, *J* = 35.6 Hz, 3H), 2.00– 1.70 (m, 2H). **^{13}C-NMR** (125 MHz, $CDCl_3$): δ 170.10, 161.72 (d, *J* = 98.99 Hz), 159.64 (d, *J* = 29.54 Hz), 150.52 (d, *J* = 4.87 Hz), 137.89 (d, *J* = 7.10 Hz), 130.26 (d, *J* = 44.27 Hz), 129.03 (d, *J* = 56.06 Hz, 2C), 128.29 (d, *J* = 20.26 Hz), 127.28 (d, *J* = 118.39 Hz, 2C), 126.21 (d, *J* =6.80 Hz, 2C), 111.68 (d, J = 53.35 Hz), 110.75 (d, J = 14.77 Hz, 2C), 58.26 (d, *J* 259.03 Hz, CH), 56.24 (d, *J* = 114.39 Hz, CH), 56.04 (d, *J* = 47 Hz, CH_{2j}), 52.3 (d, *J* = 151.49 Hz, CH2), 37.25 (*J* = 142.31 Hz, CH_2), 11.80. **LREI-MS** (70eV) m/z (% intensity): 404 (M^+,8), 216 (35), 189 (22), 144 (30), 56 (22), 43 (20).

Synthesis of compound 24.

In a 250 mL round-bottom flask, 6 mL of ethylenediamine were dissolved in 60 mL of dry DCM under nitrogen atmosphere and a solution of Boc_2O in 60 mL of DCM were added slowly with a syringe. The reaction was monitored by TLC. At the end of reaction, the organic phase was washed with 60 mL of water twice once with brine, dried over Na_2SO_4 and filtered, the DCM was evaporated in a rotatory evaporator and the product was purified by column chromatography.

Synthesis of compound 25.

In a 50 mL round-bottom flask, 647 mg of the compound **17** were placed, along with 2 drops of dry DMF and placed under nitrogen atmosphere, 10 mL of dry DCM were added, the reaction mixture was cold in an ice bath and 350 µL of oxalyl chloride were added slowly and then left under stirring for 20 minutes. Separately, a solution of 10 mL of DCM with 577µL of TEA and 510 mg of the compound **24** were prepared, and was added slowly to the reaction mixture and stirred for 30 minutes. 20 mL of water were added next, the phases were separated, and the organic phase was washed once with 20 mL of Na_2CO_3 solution, once more with water, and once with brine. The organic phase was dried over Na_2SO_4, filtered and the DCM was evaporated in a rotatory evaporator. The compound was purified by column chromatography (Hexane: AcOEt, 6:4).

Compound **25:** m.p.160-161 °C; **^{1}H-NMR** (400 MHz, DMSO-d6) δ 8.40 (t, J = 5.59 Hz, 1H) 7.60 (dd, *J* = 6.7, 2.9 Hz, 1H), 7.71 – 7.64 (m, 2H), 7.54-7.46(m, 3H), 6.85 (t, *J* = 5.56 Hz, 1H), 3.25 (dd, *J* = 12.25, 6.18 Hz, 2H), 3.08 (dd, *J* = 12.28, 6.19 Hz, 2H), 2.52 (s, 3H), 1.38 (s, 9H). **^{13}C-NMR** (400 MHz, DMSO-d6) δ 169.91, 161.99, 160.61, 156.14,

130.43, 129.18 (2C), 128.66, 128.31(2C), 113.35, 78.19, 28.69, 12.26. **LRMS** (DART+) m/z (% intensity): 346 (40, M^+), 290 (30), 246(100), 229 (13).

Synthesis of compound 26.

The method of the subsequent deprotection is described in the previous section.

Synthesis of compound 11.

In a 10 mL round-bottom flask, 272 mg of the compound **26** were placed along with 5 mL of DMF, 196 mg of the compound **13** and 308 mg of K_2CO_3. The reaction was stirred for 72 hrs. DMF was evaporated and then the crude product was dissolved in 10 mL of AcOEt, washed twice with 10 mL of water and then with 10 mL of brine. The organic phase was dried over Na_2SO_4, filtered and the solvent was removed in a rotary evaporator. The product was purified by silica gel column chromatography with DCM as mobile phase.

Compound **11**: m.p.161-162 °C; **^{1}H-NMR** (400 MHz, DMSO-d6 + $CDCl_3$) δ 8.36 (br, 1H) 8.11 (d, *J* = 2.54 Hz, 1H), 8.00 (m, 1H), 7.67 (dd, *J* = 6.89, 2.54 Hz, 2H), 7.46 - 7.37 (m, 3H), 6.81 (d, *J* = 9.23 Hz, 1H), 6.62 (br, 1H), 3.55 (dd, *J* = 11.21, 5.5 Hz ,2H), 3.46 (dd, *J* = 11.01, 5.45 Hz ,2H), 2.54 (s, 3H). **^{13}C-NMR** (400 MHz, DMSO-d6 + $CDCl_3$) δ 170.32 (d, *J* = 6.51 Hz), 163.19, 160.3, 149.66, 136.66, 130.12, 128.74 (2C), 128.43, 128.22 (2C), 125.37, 124.97, 117.56, 112.70, 109.14, 43.46, 38.45, 12.31. **LRMS** (DART+) m/z (% intensity): 403 (15, M^+ + 2), 401 (47, M^+), 333(42), 260 (100).

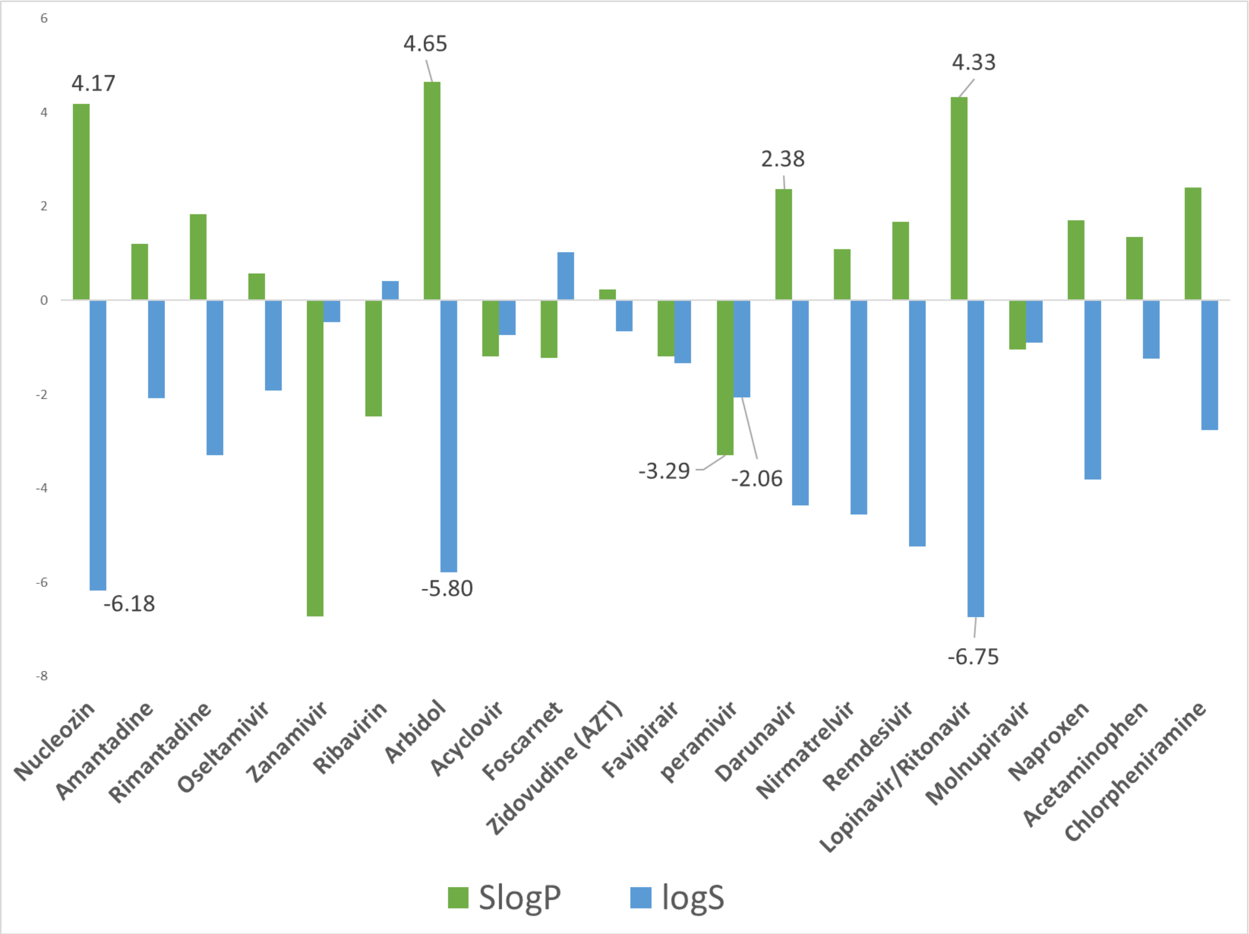

S1 Figure. The SlogP and log S calculated for nucleozin, commercial antivirals and other drugs administrated for treatment of influenza infections.

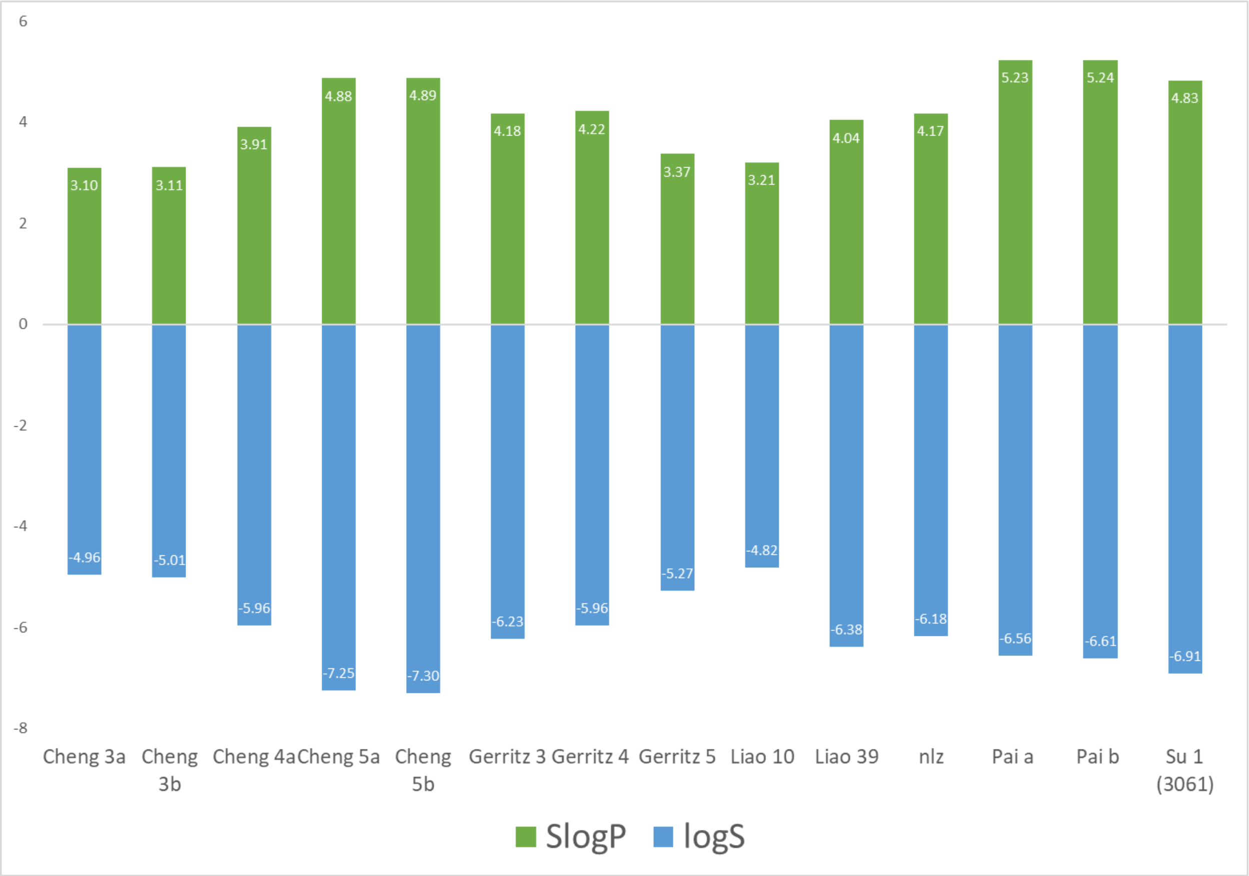

S2 Figure. The SlogP and logS calculated for nucleozin analogues. The chemical structures are shown in **S4** and **S5 Fig**.

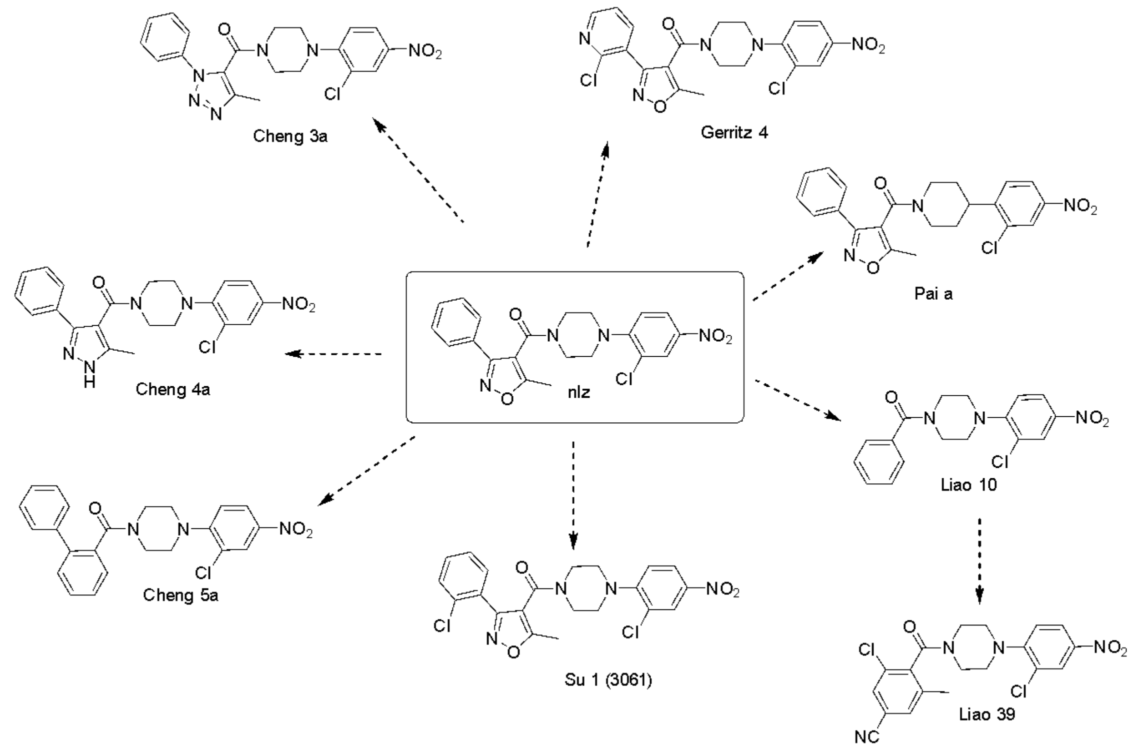

S3 Figure. Chemical structures of relevant nucleozin analogues.

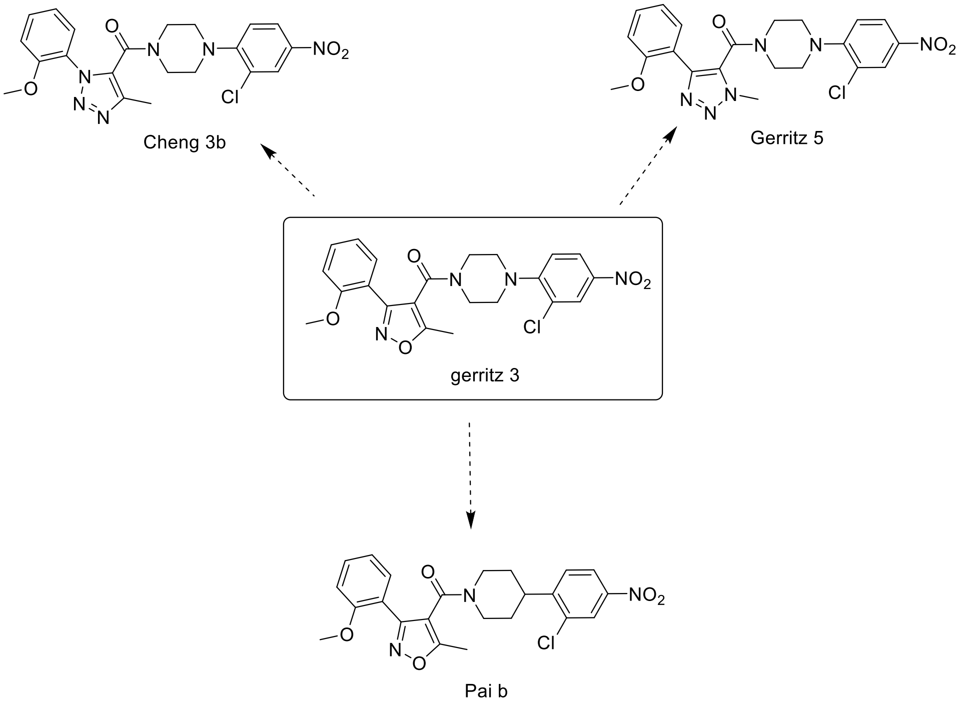

S4 Figure. Structural analogues of compound **Gerritz 3**.

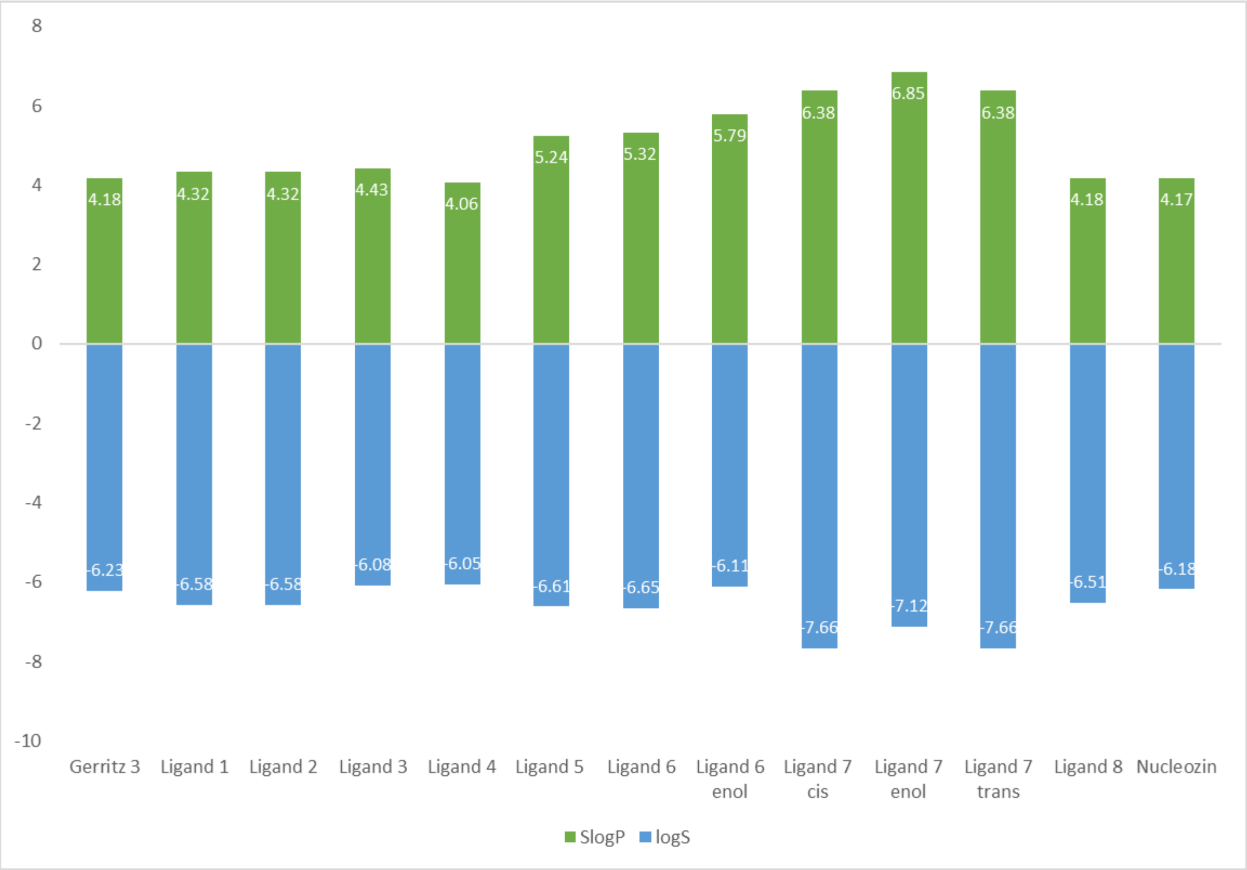

S5 Figure. SlogP and logS calculated for the analogues proposed in this work.

Characterization of **nucleozin**

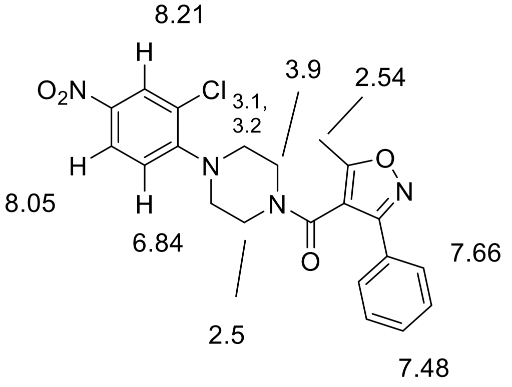

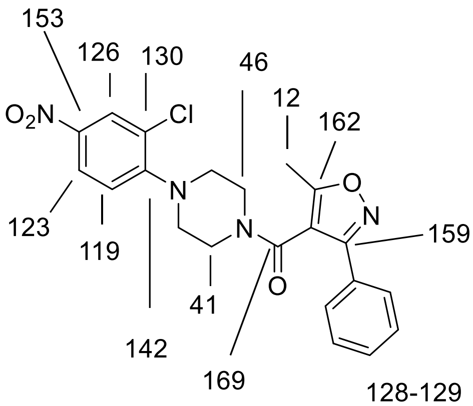

MW = 426.68 g/mol

^{1}H NMR spectrum of **nucleozin**

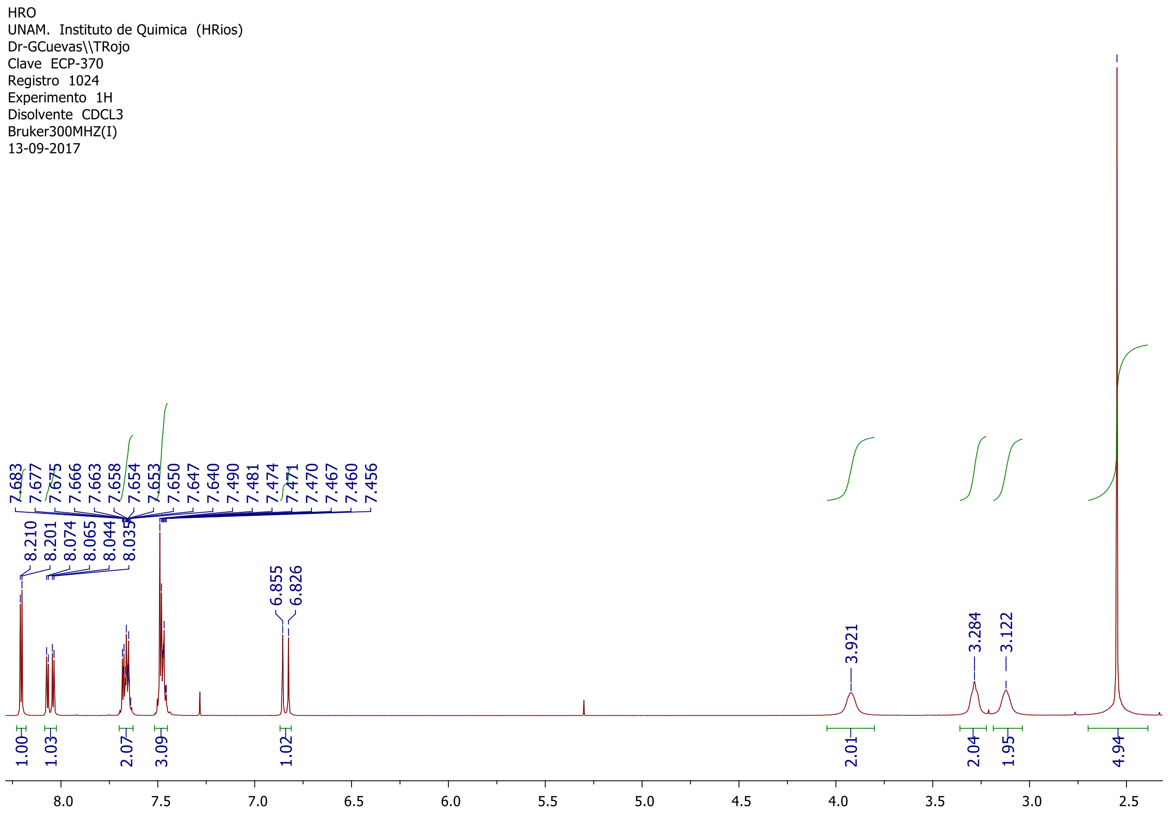

[Escriba aquí]

^{13}C NMR spectrum of **nucleozin**

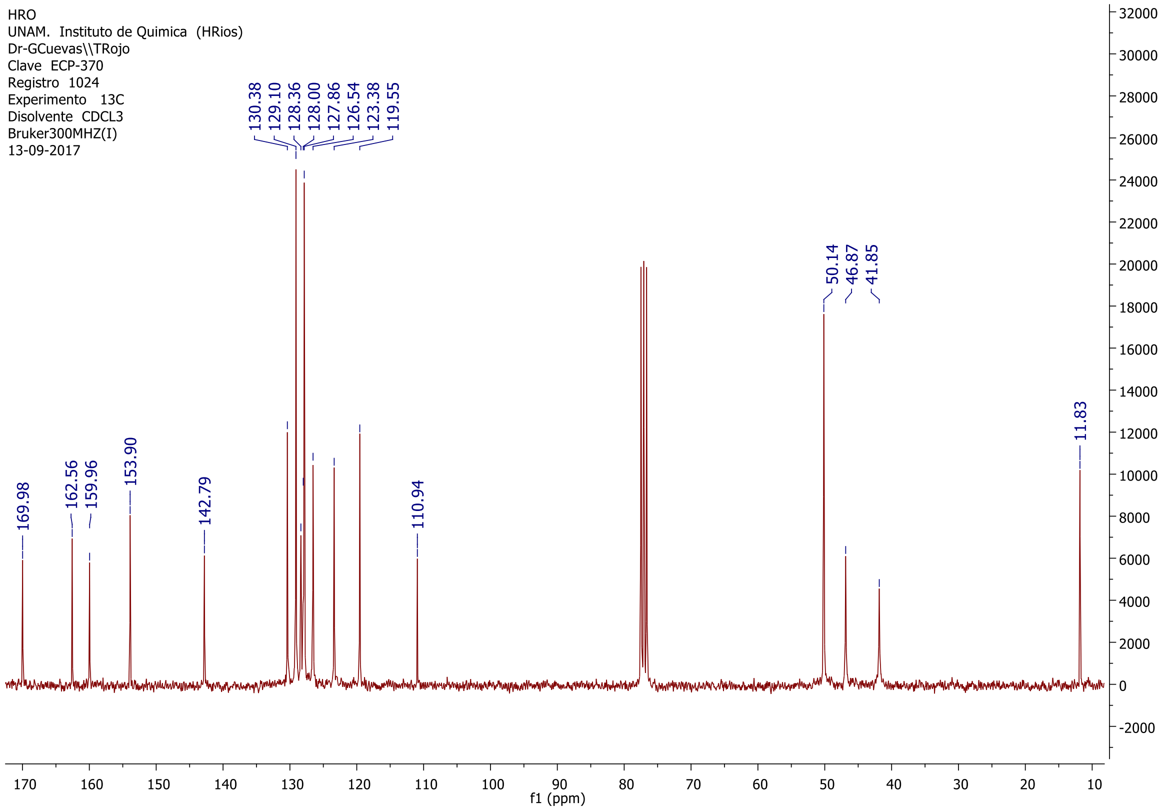

LR-DART+ Mass spectrum of **nucleozin**

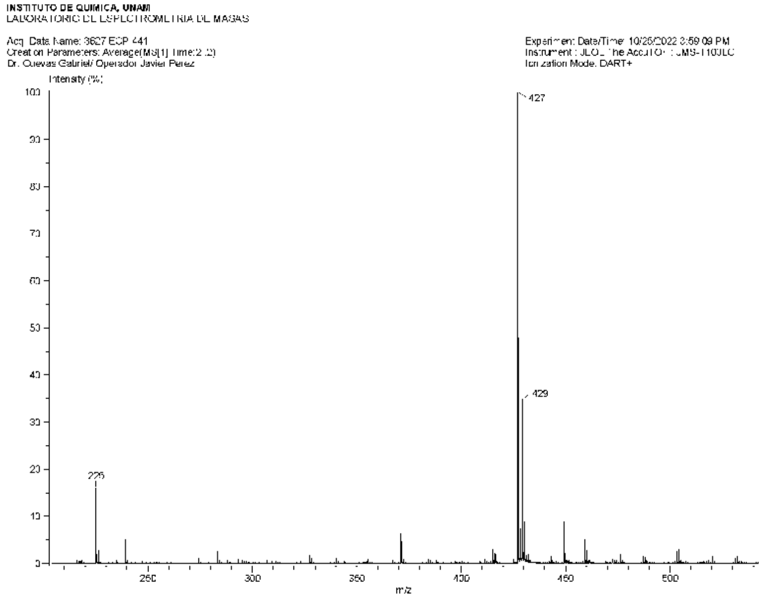

[Escriba aquí]

Characterization of the compound **20**

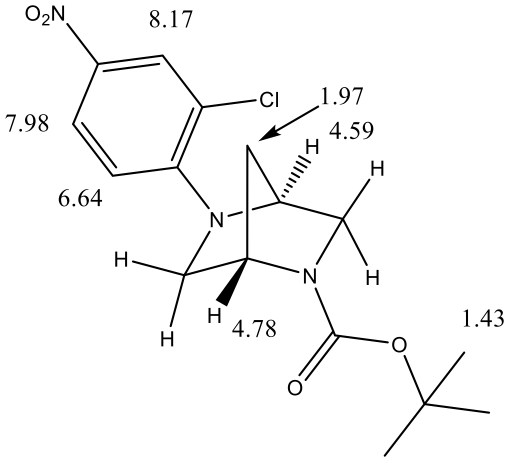

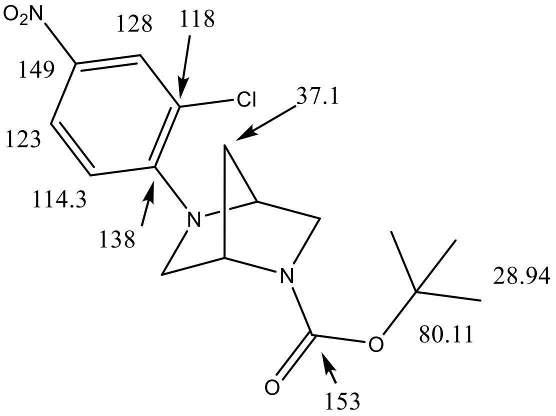

MW= 353.80 g/mol

^{1}H RMN spectra of the compound **20**

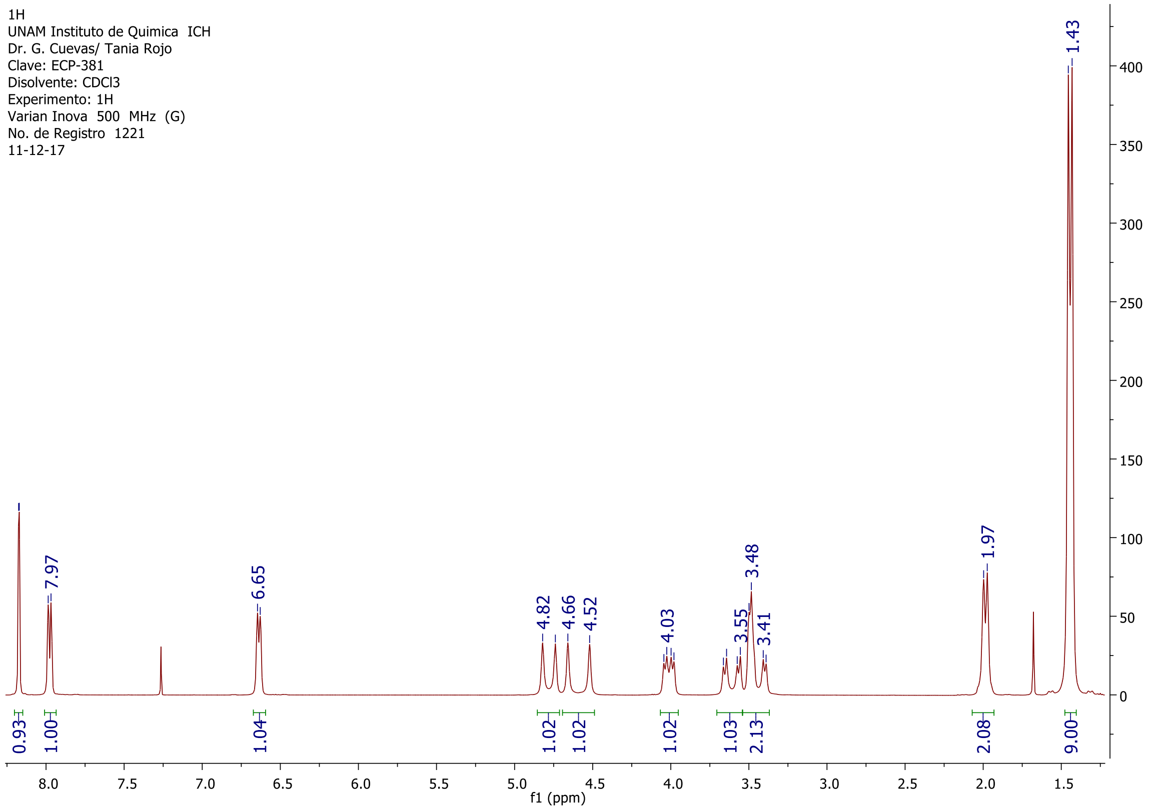

[Escriba aquí]

^{13}C NMR spectrum of compound **20**

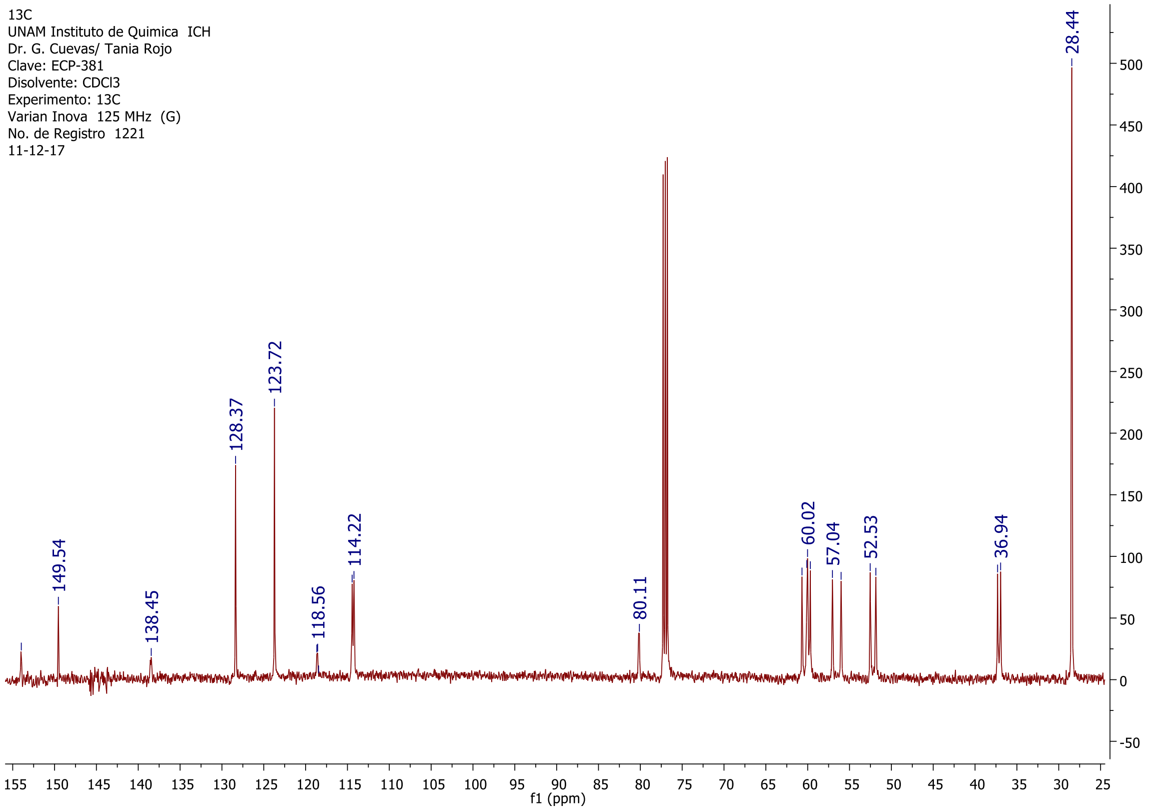

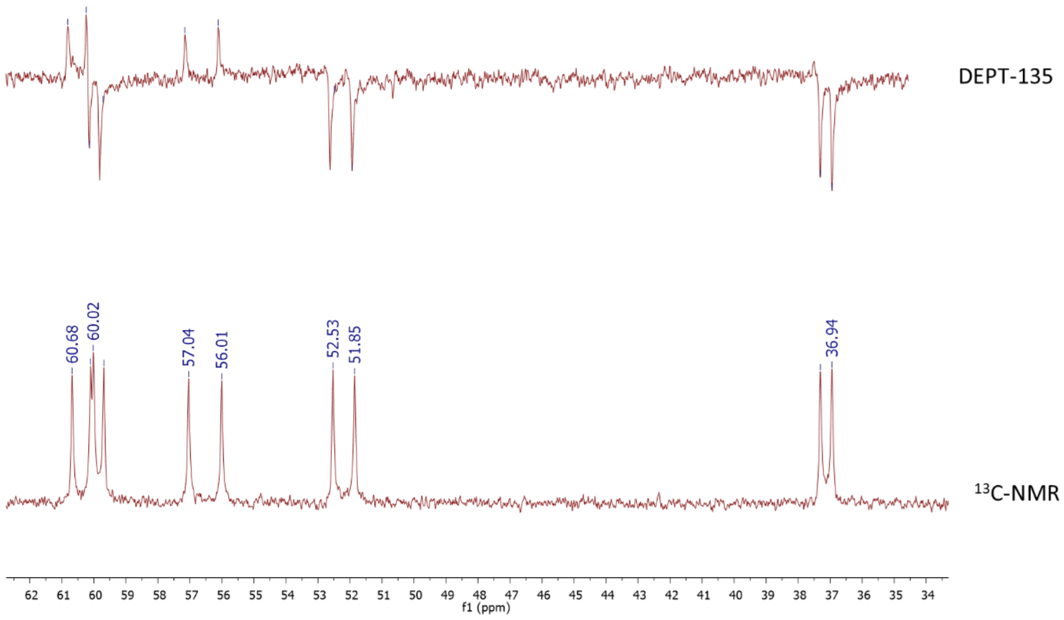

[Escriba aquí]

LR-Electronic Impact Mass spectrum of compound **20**

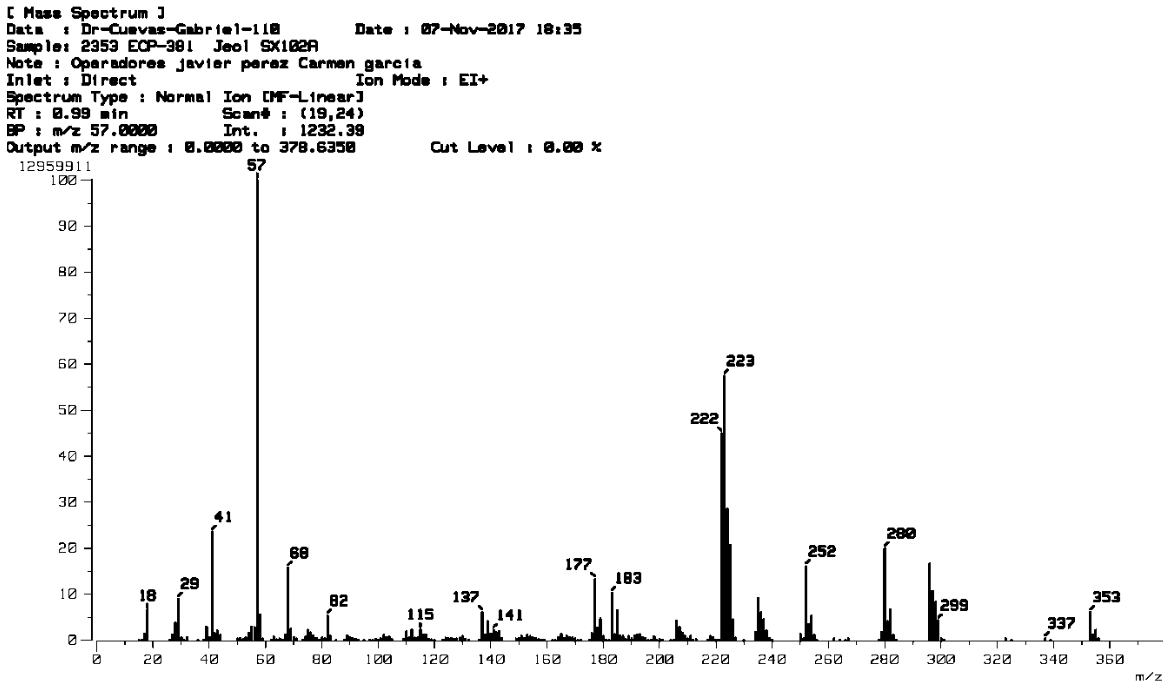

[Escriba aquí]

Characterization of compound **21**

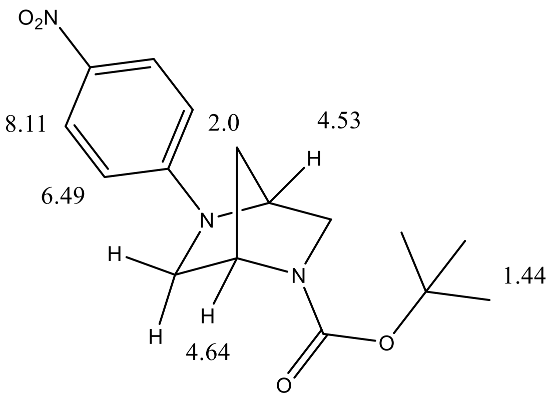

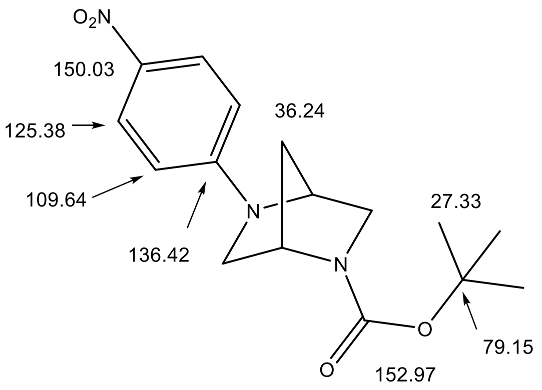

MW = 319.36 g/mol

^{1}H RMN spectra of the compound **21**

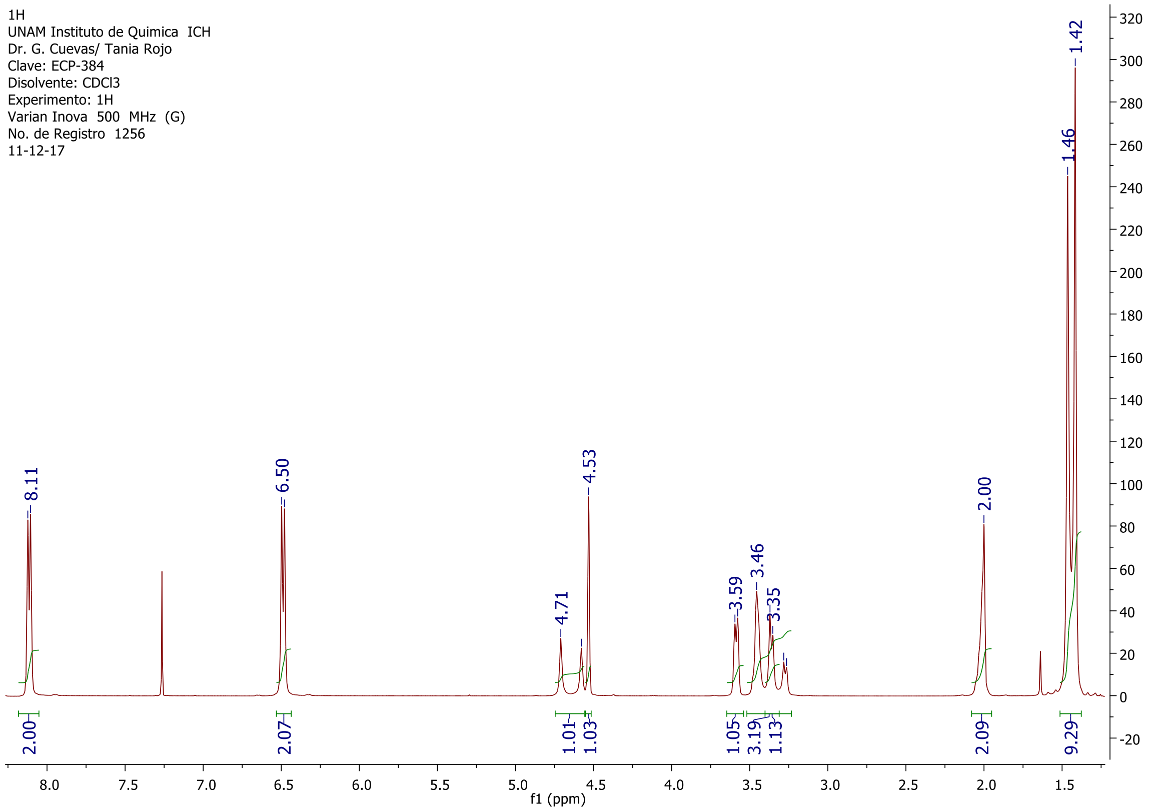

[Escriba aquí]

^{13}C NMR spectrum of compound **21**

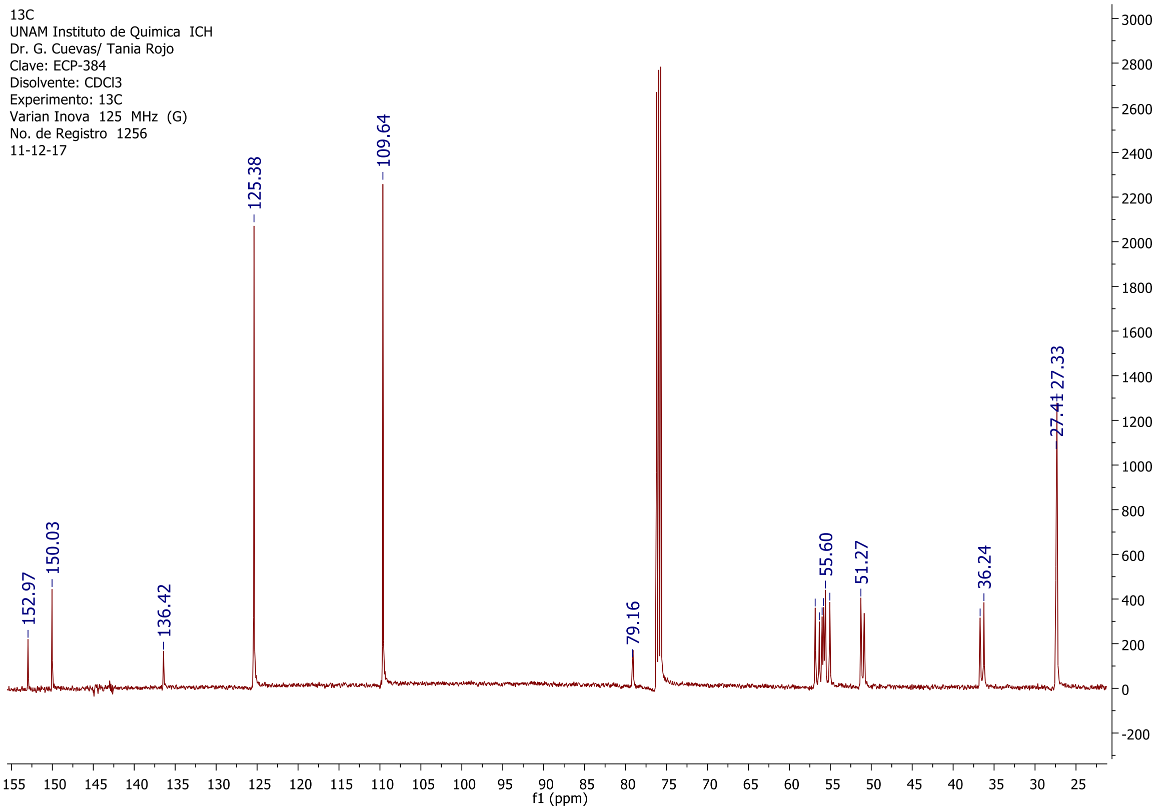

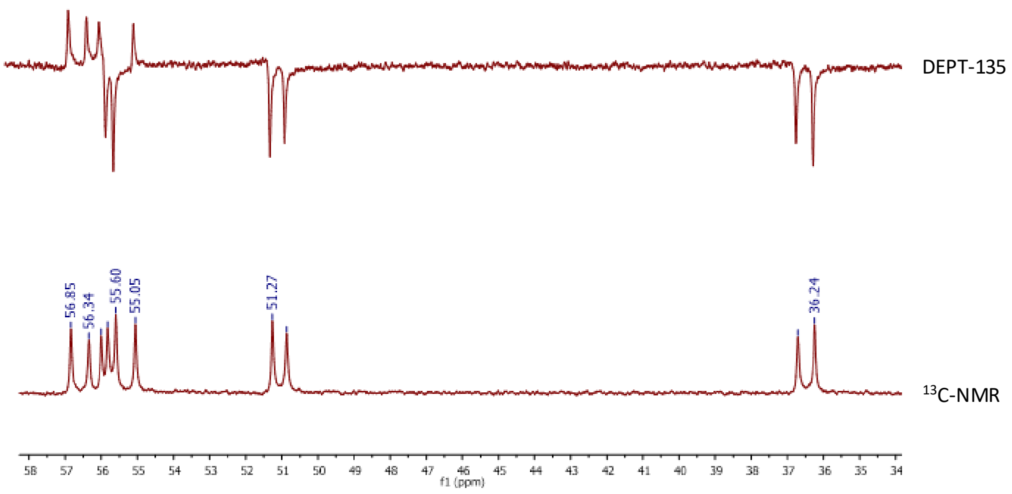

[Escriba aquí]

Characterization of compound **9**

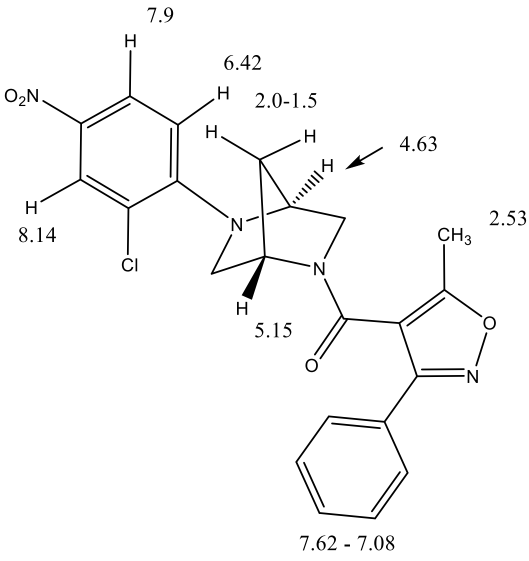

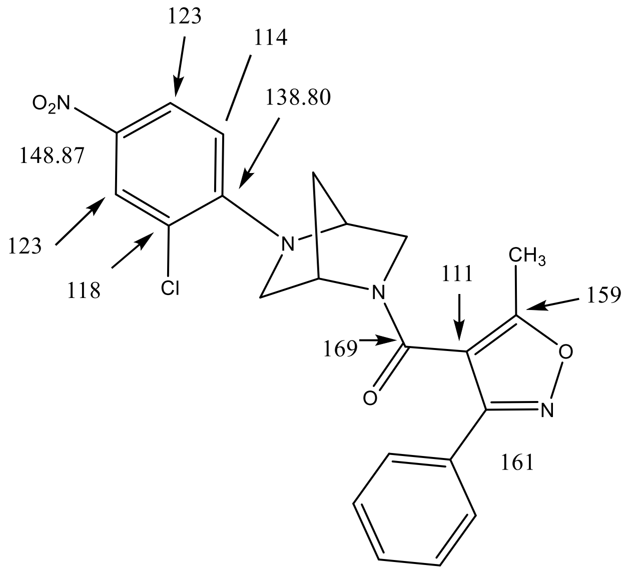

MW = 438.87 g /mol

^{1}H RMN spectra of the compound **9**

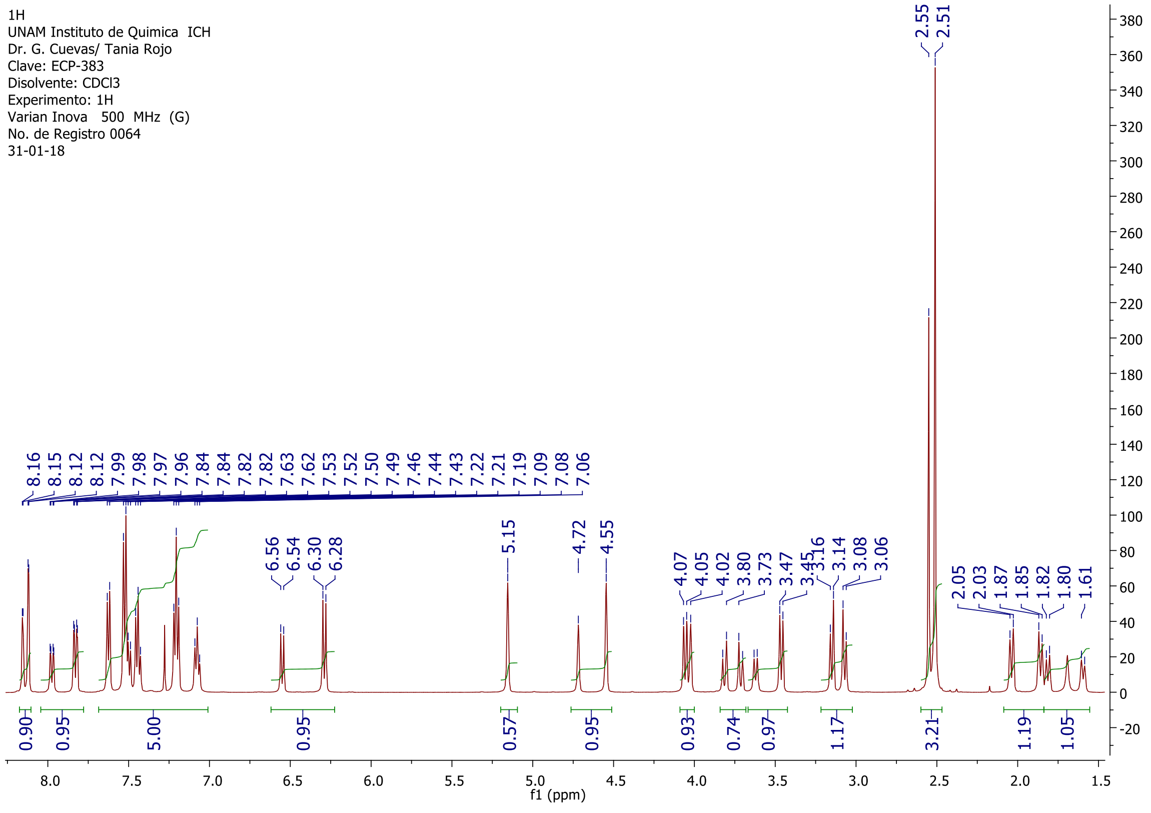

[Escriba aquí]

^{13}C NMR spectrum of compound **9**

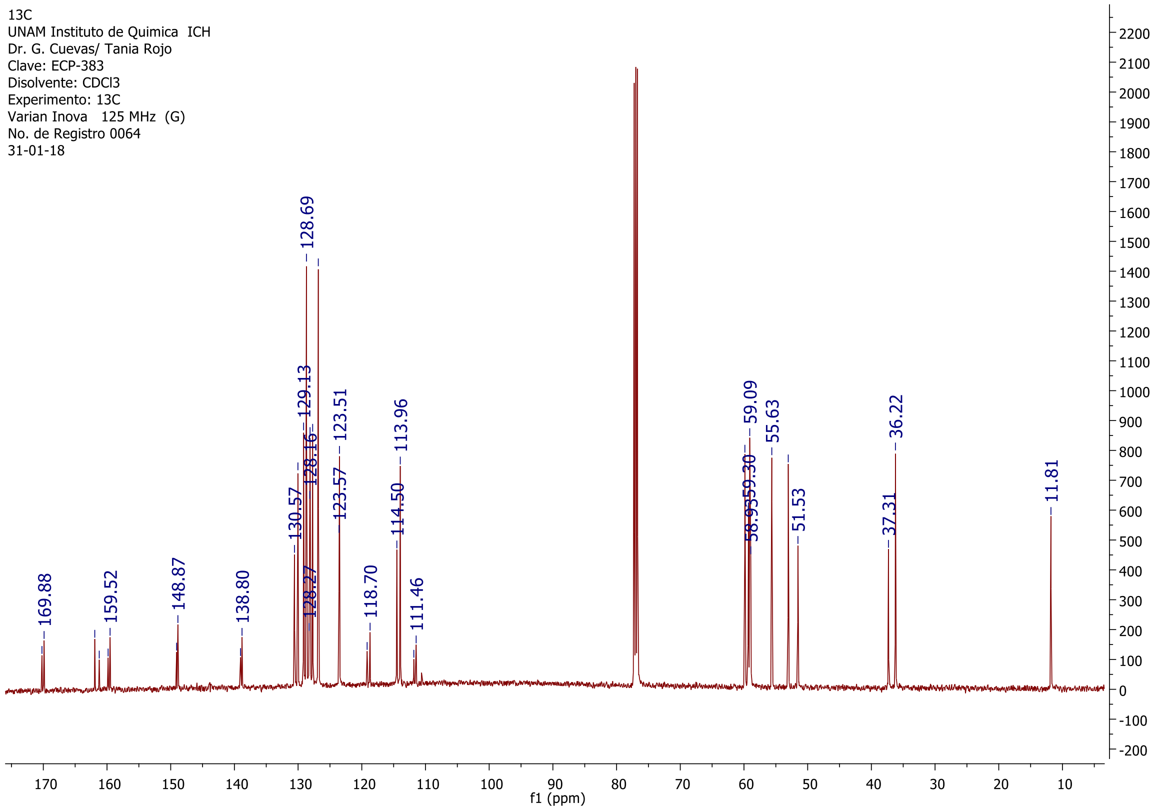

LR-DART+ Mass spectra of compound **9**

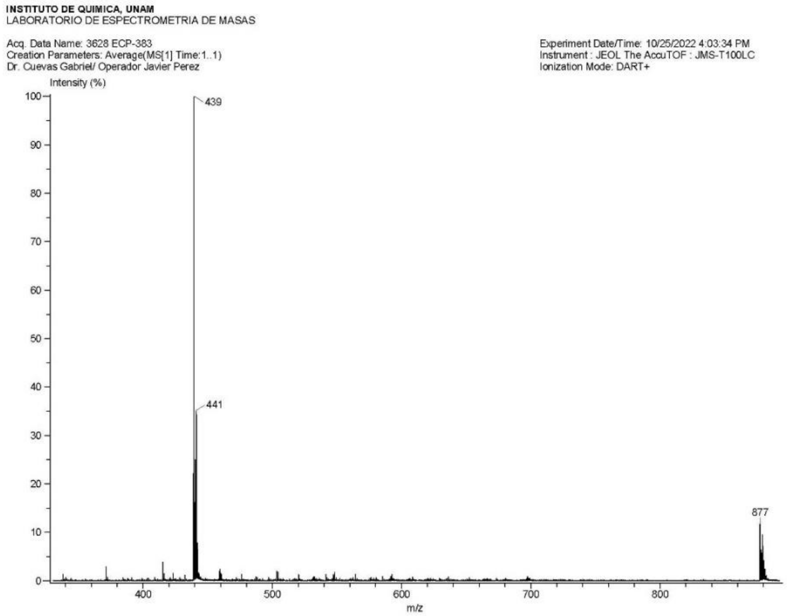

[Escriba aquí]

Characterization of compound **10**

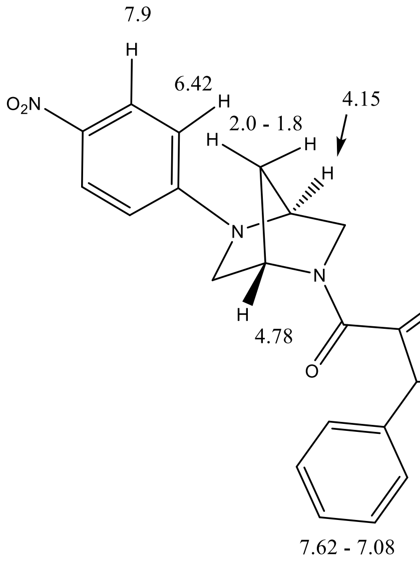

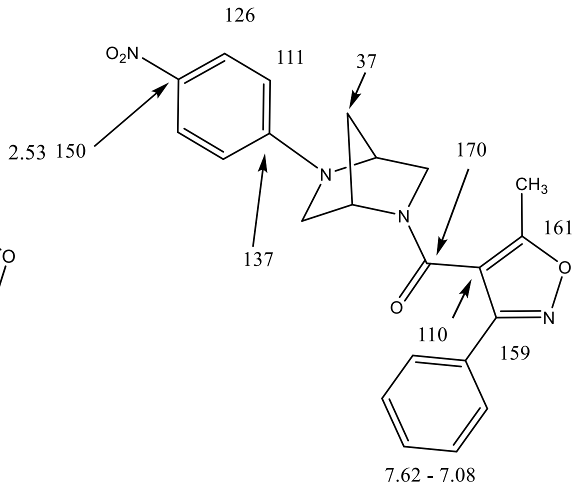

MW = 404.46 g/mol

¹H RMN spectra of the compound **10**

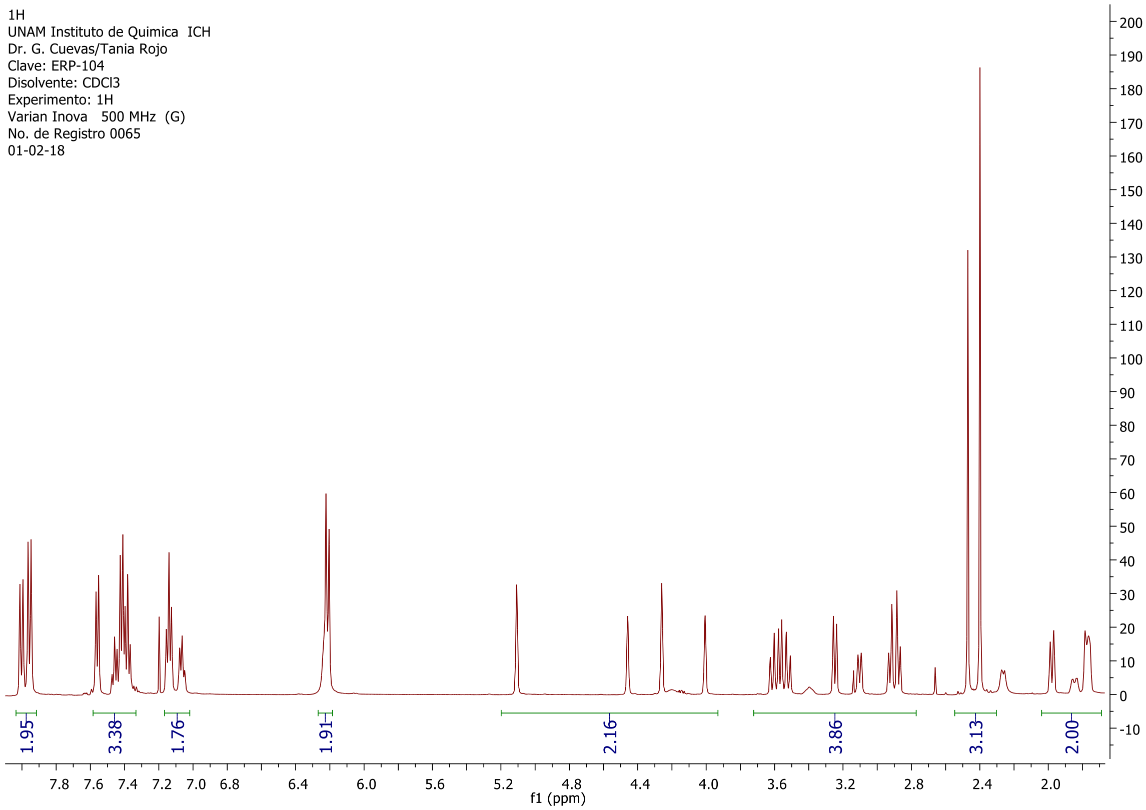

[Escriba aquí]

^{13}C NMR spectrum of compound **10**

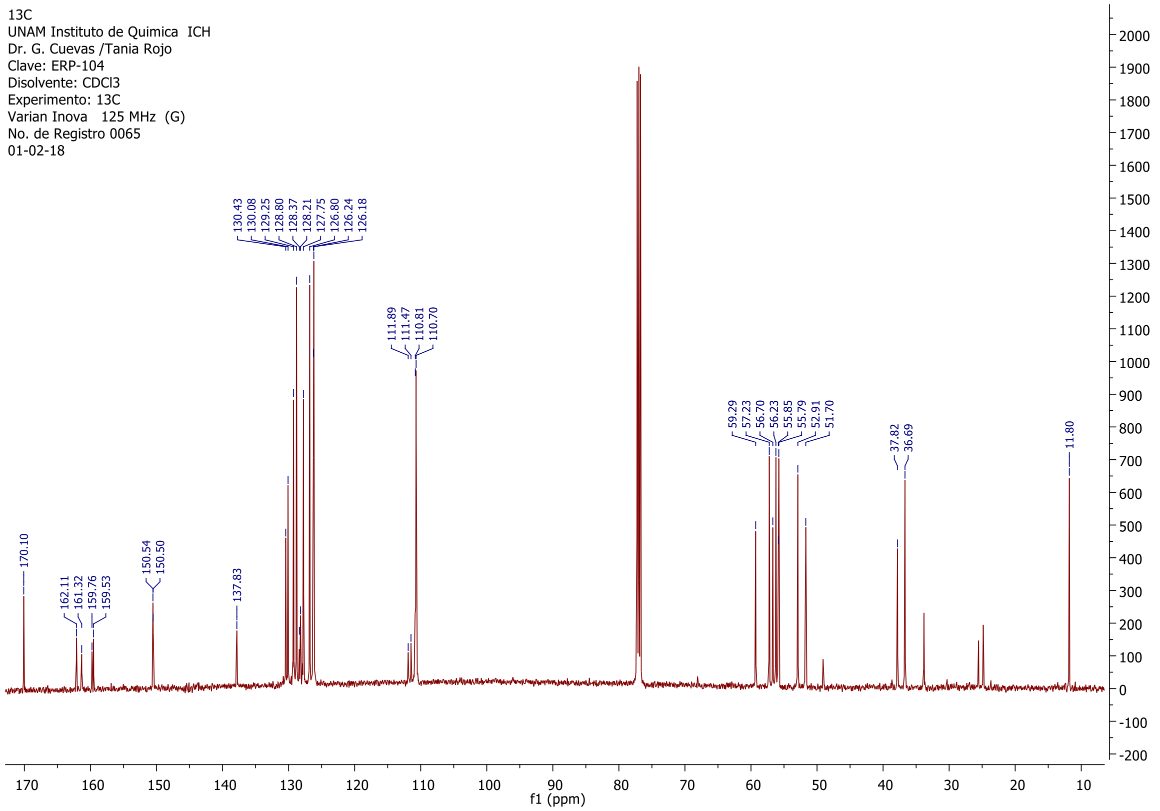

Electronic Impact Mass spectrum of compound **10**

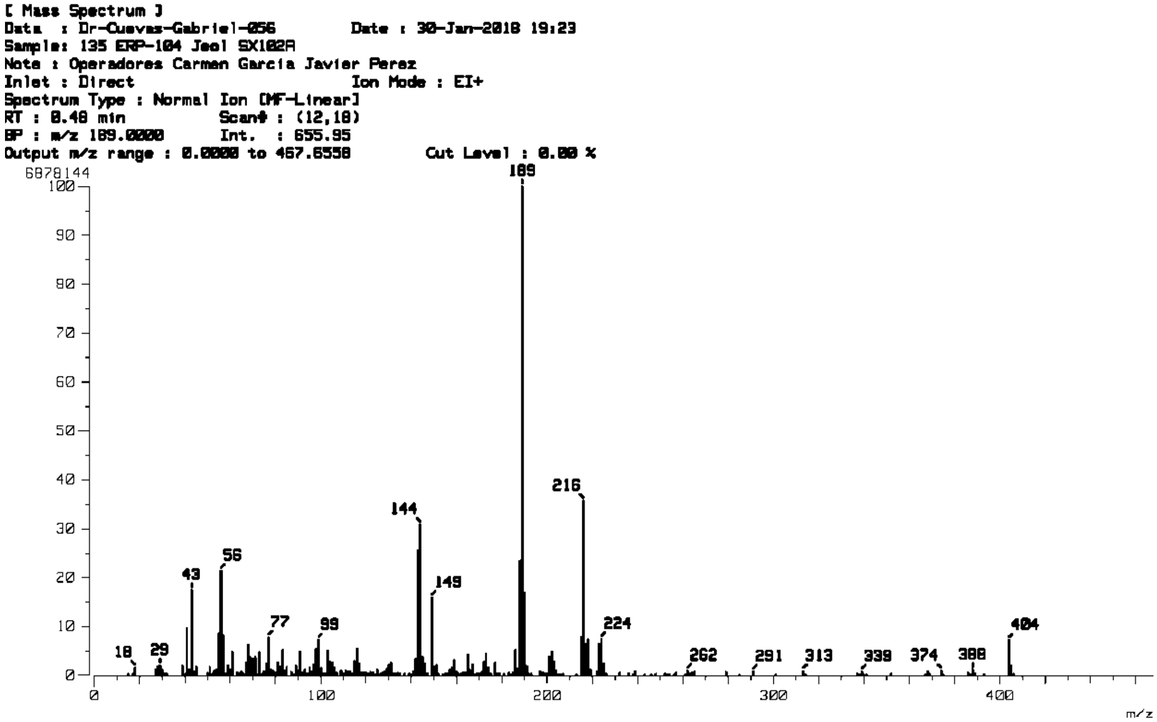

[Escriba aquí]

Characterization of compound **25**

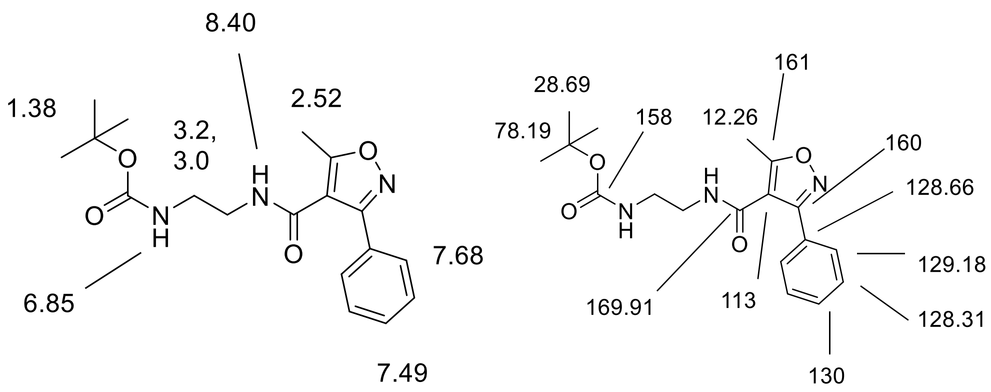

MW = 345.40 g/mol

^{1}H RMN spectra of the compound **25**

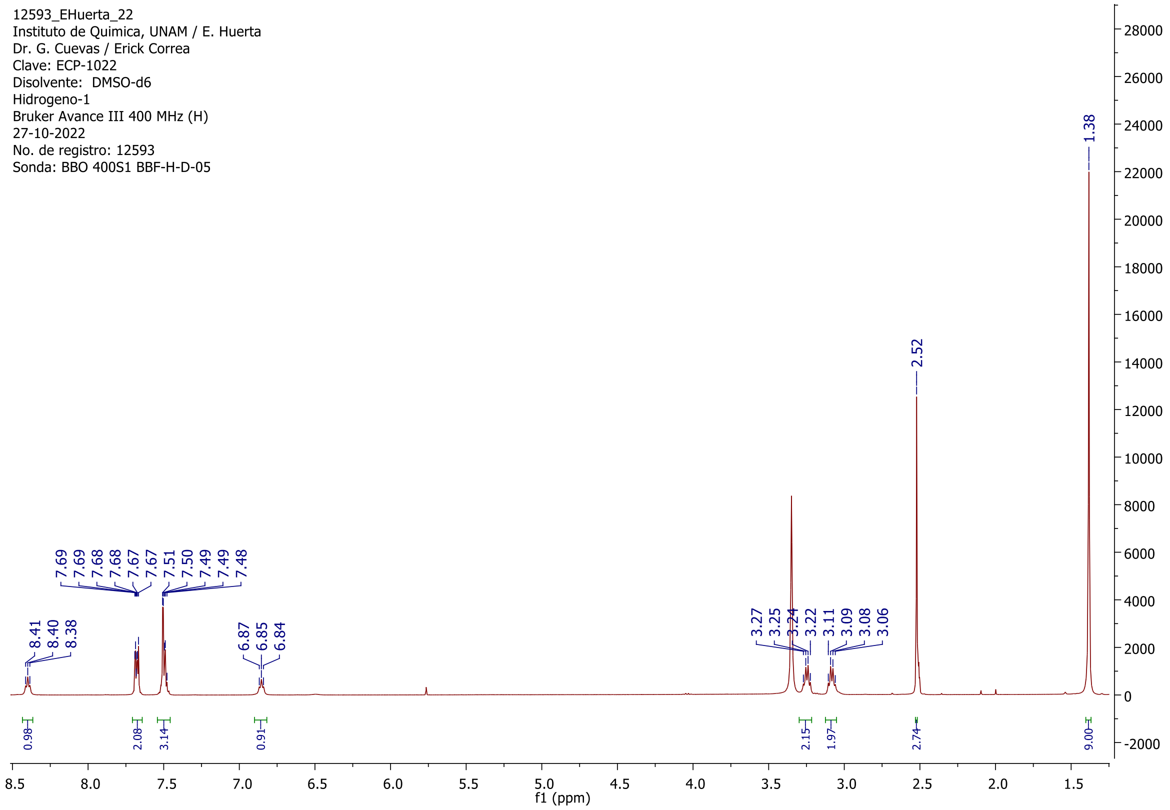

[Escriba aquí]

^{13}C NMR spectrum of compound **25**

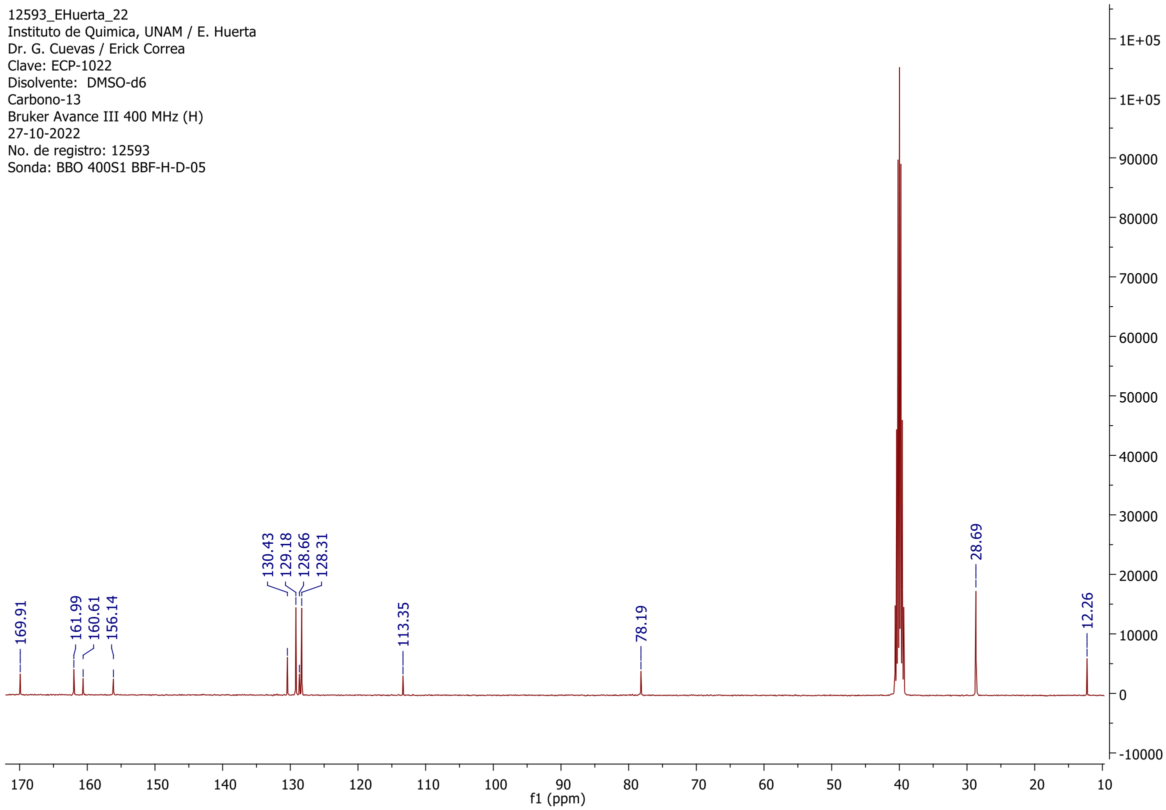

LR- DART+ Mass spectra of compound **25**

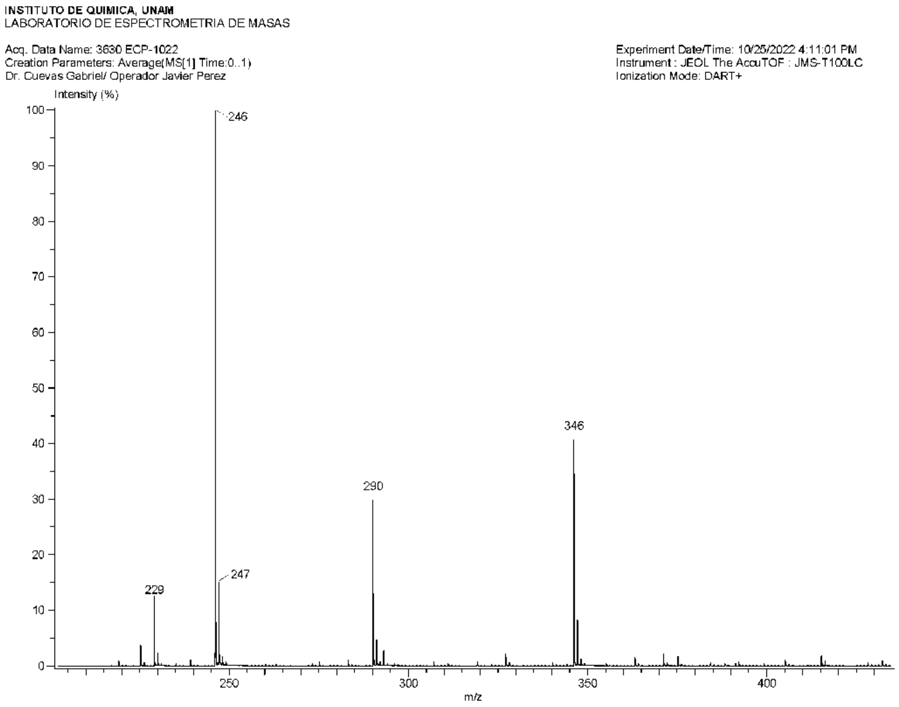

[Escriba aquí]

Characterization of the compound **11**

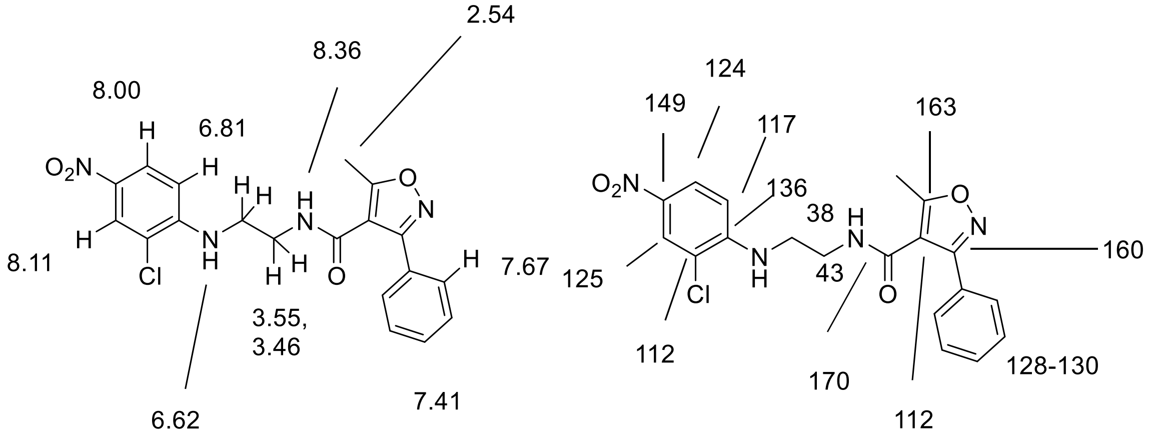

MW = 400.82 g/mol

^{1}H RMN spectra of the compound **11**

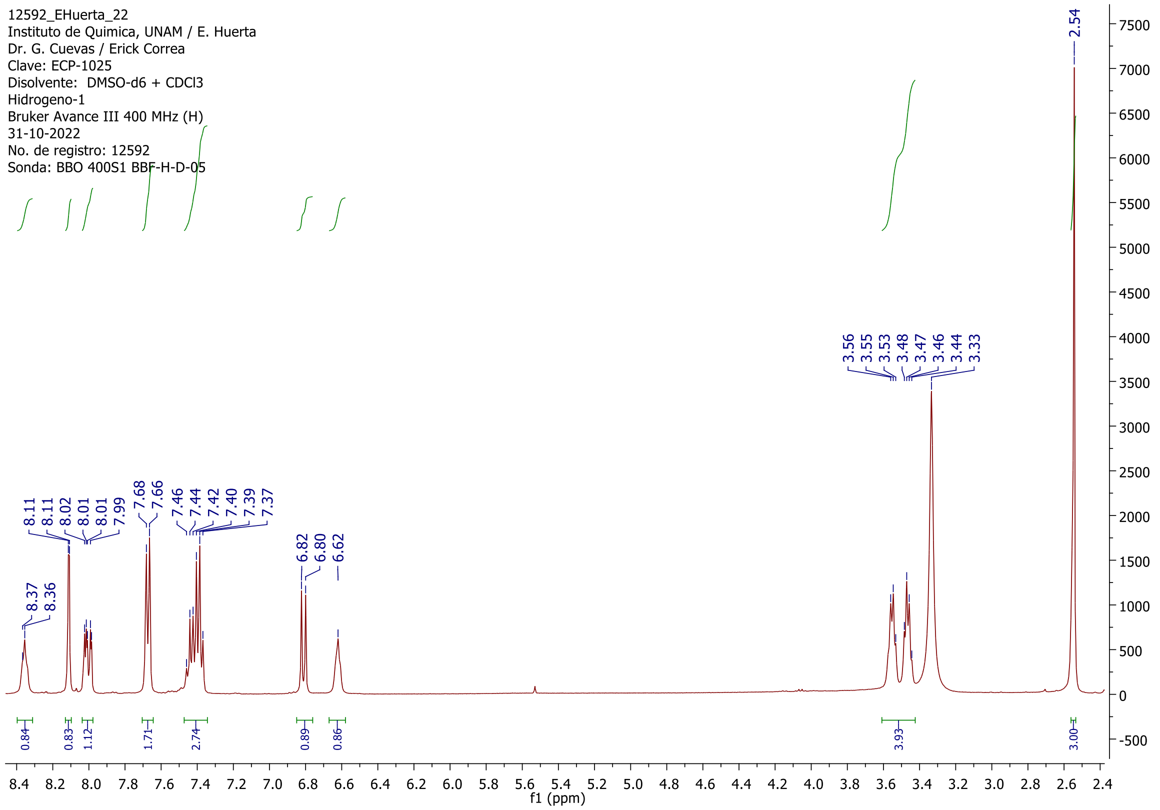

[Escriba aquí]

^{13}C NMR spectrum of the compound **11**

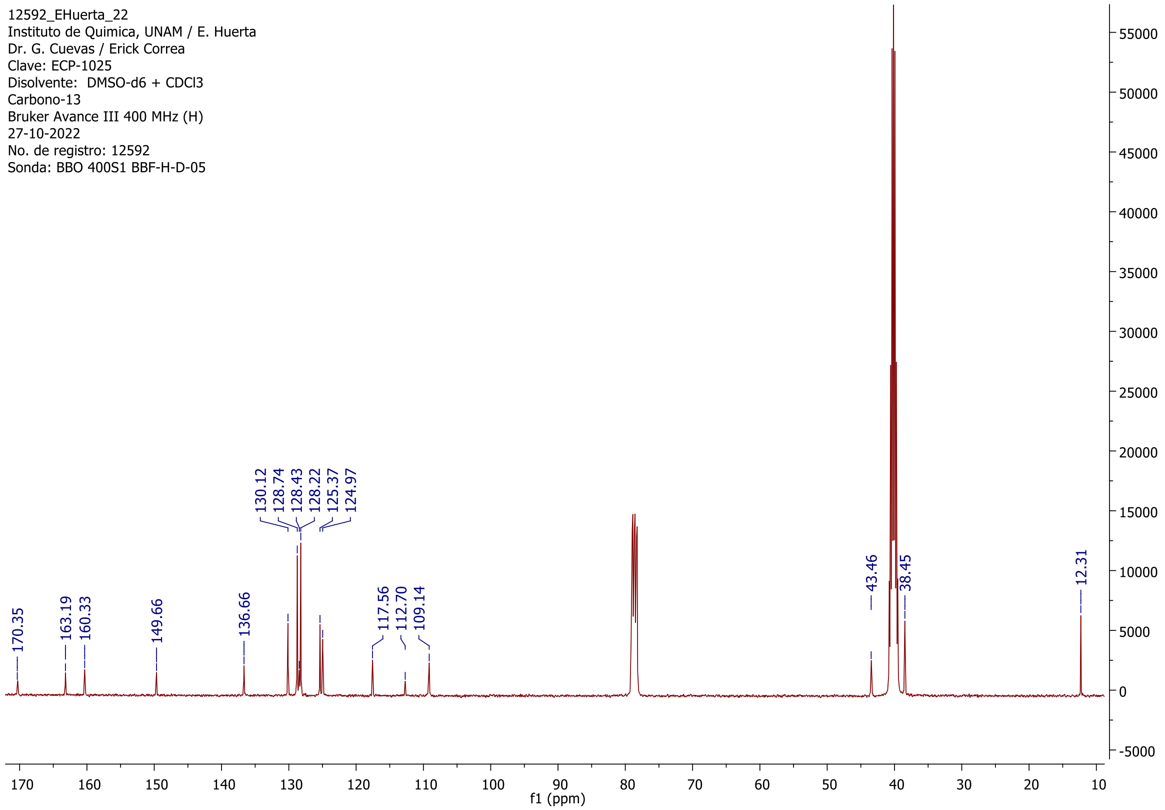

LR- DART+ Mass spectrum of compound **11**

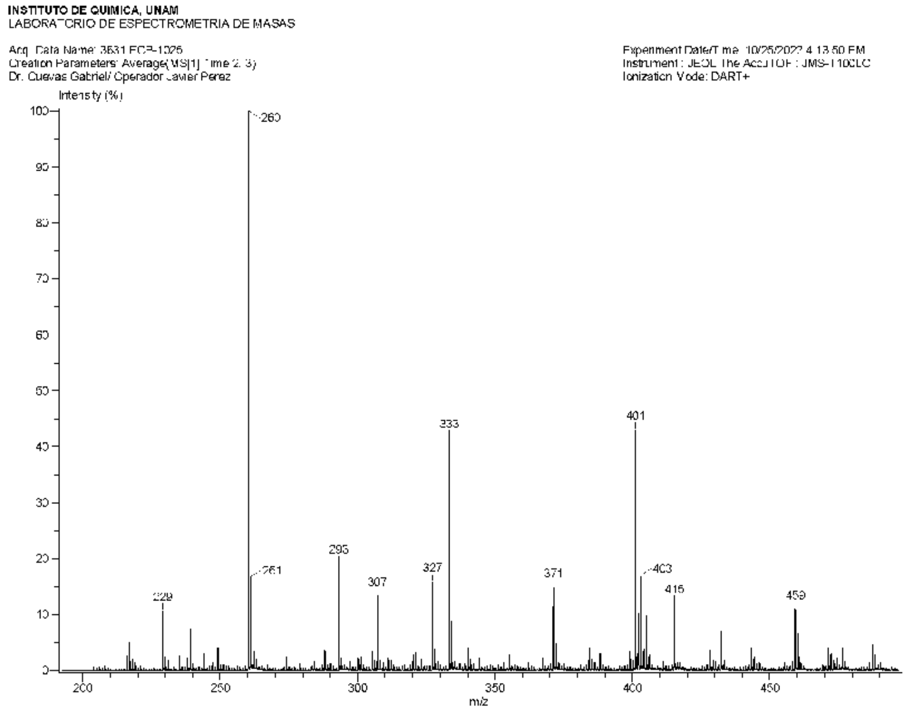

[Escriba aquí]
